# Endangered bowhead whales might buffer climate change with individual variability in movement patterns

**DOI:** 10.1038/s41598-026-36908-1

**Published:** 2026-01-27

**Authors:** Benia V. R. Nowak, Christian Lydersen, Mads Peter Heide-Jørgensen, Andrew W. Trites, Kit M. Kovacs

**Affiliations:** 1 Norwegian Polar Institute Fram Centre, Tromsø, 9296 Norway; 2 Institute for the Oceans and Fisheries, University of British Columbia, 2202 Main Mall, Vancouver, BC V6T 1Z4 Canada; 3 Greenland Institute of Natural Resources, Strandgade 91, 2, Copenhagen, 1401 Denmark

**Keywords:** Balaena mysticetus, Movement ecology, Foraging ecology, Resilience, Resource selection, Habitat use, Ecology, Ecology, Zoology

## Abstract

Assessing the vulnerability of species to global climate change and their capacity for resilience is a central challenge in ecology. Responses are variable and difficult to predict but understanding the resilience of intrinsically vulnerable species is necessary for management of natural populations. Bowhead whales (*Balaena mysticetus*) have recovered from historical over-exploitation in several Arctic regions. However, the East Greenland-Svalbard-Barents Sea (EGSB) population remains endangered, with little known about their habitat use, foraging ecology, or potential resilience. We analysed location data from 38 EGSB bowhead whales instrumented between 2017 and 2021. We performed home range analyses, fitted a modified resource selection function, and estimated move persistence to assess the influence of environmental conditions on movement patterns using linear mixed-effects modelling. EGSB bowheads used an offshore, deep-water core area year-round. Movement patterns showed considerable individual variability and suggest this population is not migratory in a classical sense, likely reducing intraspecific competition. Depth, low sea surface temperatures, and sea ice were all influential on habitat use. Both static and dynamic environmental conditions were significantly associated with apparent foraging behaviour. Although the habitat use of EGSB bowhead whales is vulnerable to continued warming, intrapopulation variability in movements might provide a buffer to climate change.

**Supplementary Information:**

The online version contains supplementary material available at 10.1038/s41598-026-36908-1.

## Introduction

 Understanding the vulnerability of species to global climate change and their capacity for resilience is a central challenge in ecology and necessary for the management of natural populations^1,2^. Responses to climate change are highly variable and can be difficult to predict given the complexity of factors at play. The vulnerability of species is influenced by a combination of intrinsic (life history strategy, physiological limitations, habitat specialisation, population size) and extrinsic (association with environmental conditions, prey availability) factors^3,4^. The vulnerability of individual populations can vary across a species’ range due to differences in population demographics as well as associations with regional environmental conditions^5^. The resilience of populations is then determined by their capacity for adaptive (genetic adaptation) and plastic (range shifts, behavioural plasticity, phenotypic plasticity) responses^6^. While r-selected species with rapid growth and reproduction may be able to adapt quickly^7^, strongly K-selected life histories with slow growth and low reproductive rates can limit the capacity or speed at which populations can adapt to environmental change, such that other strategies (when possible) become more important^8,9^. Improving our understanding of the proximate causes of vulnerability and their associated traits as well as the mechanisms that might provide resilience to buffer the effects of climate change is of considerable importance.

The bowhead whale (*Balaena mysticetus*) is the only mysticete species that is endemic to the Arctic. To cope with this extreme environment, they have evolved a number of life history, anatomical, and physiological adaptations including extreme longevity (> 200 y), advanced age at sexual maturity, a long inter-calf interval, a disproportionately large head with the longest baleen among mysticetes and no dorsal fin (which together allow them to break through thick sea ice), and the thickest blubber of any cetacean to support thermoregulation and buffer unpredictable foraging conditions^10^. While these specialised traits serve to promote fitness in an environment characterised by long-term stability, but where resources are patchily-distributed and may be seasonally or inter-annually unpredictable, they may be maladaptive under long-term unidirectional climate change, limiting the bowhead whale’s potential to adapt^11^. This species occurs in four geographically and genetically distinct populations, all of which underwent intensive commercial exploitation. While the Bering-Chukchi-Beaufort Sea (BCB) and East Canada-West Greenland (ECWG) populations have since recovered, the Sea of Okhotsk (OKS) and East Greenland-Svalbard-Barents Sea (EGSB) populations, which were reduced to levels where they were considered functionally extinct, remain endangered^12,13^.

The EGSB population is estimated to have been the largest bowhead whale population historically, but was subjected to both longer and more intensive harvesting than any of the other populations^14^. Partial surveys over different parts of their range have estimated that the population likely numbers in the low hundreds^12,15^. Due to their small population size and proclivity for dense sea ice, relatively little is known about their basic ecology. However, recent studies with small sample sizes suggest that while individuals from this population show a strong preference for cold, ice-covered waters like other bowhead whales, their movement patterns appear to be atypical compared to the seasonal migrations performed by other populations, following the formation and recession of sea ice and zooplankton distributions^16,17^. This small population of bowhead whales provides an opportunity to identify sources of vulnerability to climate change that contribute to interpopulation variability in one of the most K-selected species on the planet^18^, as well as insight into potential mechanisms of resilience for this and other habitat specialists facing similar pressures.

**Fig. 1:**
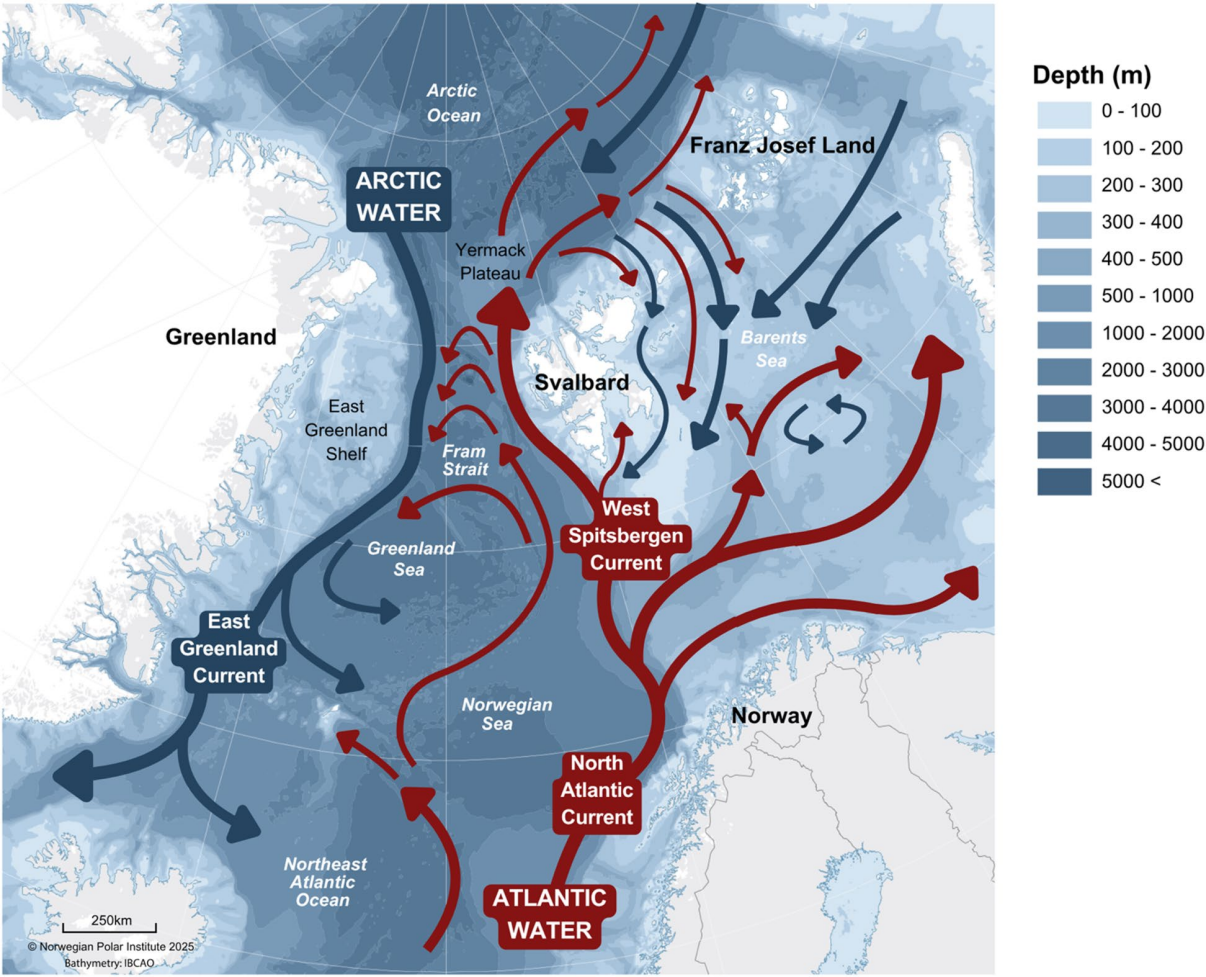
Regional map of EGSB bowhead whale habitat including Greenland, the East Greenland Shelf (EGS), the Yermack Plateau, Svalbard, and Franz Josef Land (FJL). Includes bathymetry and circulation patterns of the Fram Strait and Barents Sea with major pathways of Arctic Water (in blue, the East Greenland Current (EGC)) and Atlantic Water (in red, the North Atlantic Current (NAC) and West Spitsbergen Current (WSC)) transport.

The Arctic is warming rapidly with marked declines in the seasonal duration and extent of sea ice^19,20^. Although these changes are impacting the habitat use of all bowhead populations (along with other endemic and seasonally-resident marine species) the environment inhabited by the EGSB population is changing the fastest and with the greatest intensity. This region is influenced by two major current systems (Fig. 1)^21^. The West Spitsbergen Current (WSC) is the primary heat source for the Arctic Ocean and together with the East Greenland Current (EGC) is responsible for 90% of heat exchange that occurs in the Arctic^22^. Recent increases in the volume and temperature of incoming Atlantic Water (referred to as “Atlantification”) are amplifying warming of the Arctic and sea ice losses^23–25^. The effects of this are most pronounced at the interface between Atlantic Water and Arctic Water in the Fram Strait (Fig. 1), within the habitat of EGSB bowhead whales^26^. Over recent decades, Atlantification has led to considerable shifts in vertical stratification and horizontal gradients of water masses with consequent changes to hydrography and circulation^27,28^.

The physical effects of Atlantification have also altered the biological conditions of this habitat. While the biomass of calanoid copepods (the preferred prey of bowhead whales) on the East Greenland Shelf (EGS) has been dominated by Arctic species (*Calanus glacialis*, and also *C. hyperboreus*), the boreal *C. finmarchicus* is increasingly advected into this region, with possible local recruitment^29–31^. Obtaining data on the distribution of productivity in this region is extremely difficult due to the presence of heavy drift ice and a prolonged polar night but is nevertheless important for assessing responses to climate change at the ecosystem level.

Data on the relationships between bowhead whales and their environments are needed across the species’ range to understand population differences in vulnerability to climate change and how they may respond to a changing Arctic. In this paper, we first assess the population-level distribution of EGSB bowhead whales using movement data derived from satellite-linked data transmitters (SLDTs). We then use a modified resource selection function to quantify the influence of broad-scale environmental conditions on habitat use. Finally, we explore the influence of static and dynamic environmental conditions that likely shape the spatio-temporal distribution of productivity on the movement patterns of EGSB bowhead whales.

## Methods

### Bowhead whale data collection

**Fig. 2:**
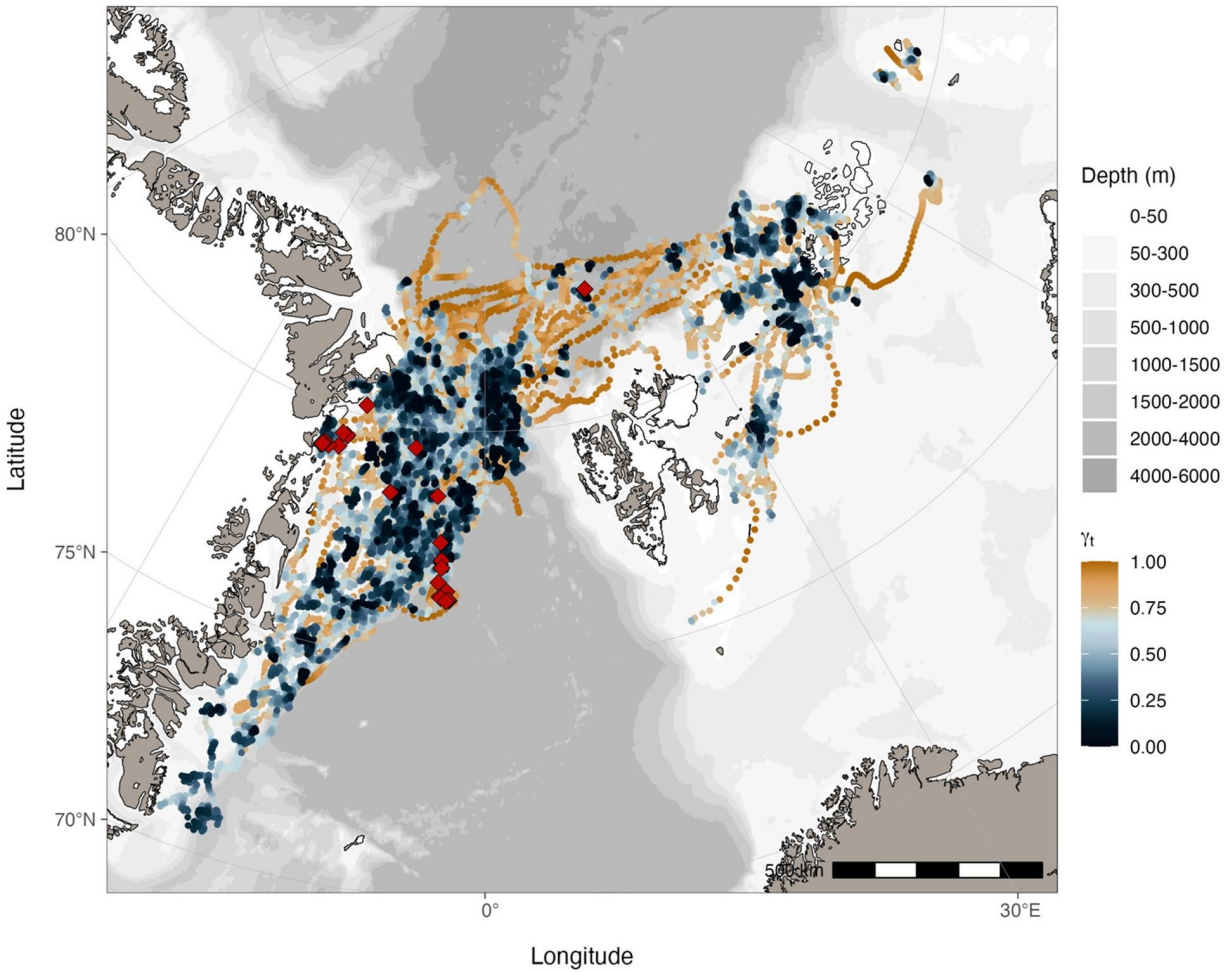
Locations of EGSB bowhead whales instrumented in the Fram Strait between 2017 and 2021 (*n* = 37). Locations were predicted using random walk and correlated random walk state-space models at a four-hour time step and re-routed around land. Locations are mapped using the Arctic Polar Stereographic projection and the scale bar included for reference is accurate at 71ºN. Locations are coloured by move persistence estimates (γ_t_) normalized across individuals to emphasize differences in move persistence within individual tracks. Instrument deployment locations are included as red diamonds. The map was produced using the R package *ggOceanMaps*^33^.

Between 2017 and 2021, 38 EGSB bowhead whales were instrumented with transdermal SLDTs (SPOT-303/372 or SPOT-177P and SPLASH10-302/373, referred to as SPOT and SPLASH tags, respectively) Wildlife Computers, www.wildlifecomputers.com; Table 1; see Supplementary Information for data collection details). One individual (GW19-01) was instrumented with two SPOT tags (the second tag was deployed because the first tag did not fully enter the blubber) and one SPLASH10-F-333 Low Impact Minimally Percutaneous Electronic Transmitter (referred to as LIMPET tag for programming distinction) for performance comparisons between tag attachment types and to collect fine-scale location data (Table S1). SLDTs were deployed in late summer/early fall in the Fram Strait or north of Svalbard (Figs. 1 and 2). In addition, skin biopsies were collected from 19 of the instrumented individuals to genetically determine sex.

**Table 1:** Deployment record and location data summary for EGSB bowhead whales instrumented in the Fram Strait between 2017 and 2021. Predicted locations include data from overlapping dates for individual GW19-01.

Deployment Year	Deployment Months	SLDT Model	Instrumented Individuals	Transmitted Locations	State-Space Modelling			
					Individuals	Track Segments	Fitted Locations	Predicted Locations
2017	May/Jun	SPOT	13	16,374	13	26	12,325	6,066
2018	Aug/Sep	SPOT	9	12,704	8	20	9,416	6,440
2019	Sep	SPOT, LIMPET	1	2,259	1	3	1,686	966
2020	Sep	SPLASH	2	7,758	2	5	6,199	1,910
2021	Aug	SPLASH	13	46,175	13	32	34,618	12,328
**Total**			**38**	**85**,**270**	37	86	64,244	27,768

Animal handling protocols were approved by the Norwegian Animal Research Authority (FOTS ID: 26614) and the Governor of Svalbard (Sysselmannen Permit ID: 21/01197-2). Research was conducted in accordance with the relevant guidelines and regulations. We have conducted and reported the research according to the ARRIVE guidelines relevant for research on wild animals.

### Location data processing

Data processing and analyses were performed using the statistical software R version 4.4.0^32^ and maps (excluding Fig. 1) were produced using the R package *ggOceanMaps* version 2.2.0^*3*3^ which utilised land and glacier polygons from Natural Earth Data^34^ and bathymetry from NOAA^35^. To account for location uncertainty and estimate locations along movement tracks, we fitted continuous-time state-space models using the R package *aniMotum* version 1.2–06^36^. These models can be fitted using a combination of both Argos and Fastloc GPS location data. This package also allows for the estimation of time-varying move persistence along tracks within the same modelling framework. Given the geographic distribution of locations, coordinates were projected to the Arctic Polar Stereographic projection to ensure appropriate calculation of movement metrics. Prior to analysis, location data were automatically and manually filtered for quality control and tracks (including separate tracks for GW19-01) were separated into track segments to improve state-space model convergence, reduce looping artefacts, and exclude estimates where predicted locations would have high standard errors (Supplementary Information).

To obtain the best possible estimates for true locations along track segments, we fitted both random walk and correlated random walk state-space models to each track segment with a prediction time step of four hours and selected the model that provided the best model fit^36^. Locations within track segments were predicted at a regularized time step to approximately normalize the number of locations per day (mitigate clustering and fill temporal gaps) and reduce spatio-temporal autocorrelation. Numerous iterations of different filtering criteria (i.e., permissible data gaps and track segment durations) were attempted prior to model fitting, however no combination provided good model fits using just one model type (random walk or correlated random walk) for all track segments. Most track segments, particularly those that were long with small time intervals between locations, fit best using a random walk. Fitting these tracks using a correlated random walk model often resulted in over-smoothing.

 Model validation was performed by visual inspection of both one-dimensional (time-series) and two-dimensional (geographic coordinate) plots of predicted standard error estimates to detect lack of fit, including over-smoothing and looping artefacts present in predicted compared to observed locations. We conducted prediction residual diagnostics using One-Step-Ahead Residuals (OSAR) including time-series, Q-Q plots, and autocorrelation function (ACF) plots and comparison of Corrected Akaike Information Criterion (AICc). We also assessed track uncertainty from 100 replicate tracks simulated from the joint precision matrix of each state-space model fit. Track segments with fewer than 54 locations (the shortest track segment without convergence or model fit issues) after filtering were removed from the dataset. Predicted locations were re-routed around land using a map scale of 1:10 m and a buffer of 10,000 m to avoid re-routing locations occurring within fjords in eastern Greenland or within the Franz Josef Land (FJL) archipelago. Locations from individual tags on GW19-01 were modelled separately and then truncated, as tracks with temporal overlap showed consistent deviations in geographic coordinates. Locations at the start of track 1 were truncated to the end of track 2 (the LIMPET tag) to preserve the highest quality locations (Table S1). Additionally, locations at the end of track 1 were truncated to the start of track 3 (the longest track) to preserve locations predicted with the best model fit.

### Home range estimation

To explore the spatial distribution and home range of EGSB bowhead whales instrumented in our study, we used the R package *adehabitatHR* version 0.4.21 to estimate the population utilization distribution using the kernel density method^37^. Bandwidth selection by least-squares cross validation failed to converge so we used a visual ad-hoc method for selecting bandwidth to prevent over- or under-smoothing common in kernel density estimation. We first calculated the reference bandwidth (h_ref_) using the ad-hoc method of estimating the smoothing parameter and then sequentially reduced h_ref_ by increments of 0.1 until 0.1h_ref_. We then chose the smallest increment of h_ref_ for the bandwidth until the otherwise contiguous home range (aside from small separated areas initially present) began to separate^38,39^. The home range (95%), core area (50%), and hotspots (25%) were then estimated for predicted locations using the bandwidth 0.6 h_ref_ over a grid size of 8,000 m. We visually and statistically (using Wilcoxon Rank Sum tests) assessed how the oceanographic conditions that influence circulation patterns (including eddy kinetic energy (m^2^ s^− 2^), sea ice concentration, depth (m), slope (º), barotropic streamfunction (m^3^ s^− 1^), and sea surface height (m)) at locations within the largest hotspot located in the central Fram Strait differed from locations outside this hotspot (Supplementary Information).

To assess the degree of overlap in home ranges and core areas between seasons (spring = March, April, May, summer = June, July, August, fall = September, October, November; winter = December, January, February), we calculated the Bhattacharyya’s affinity (the joint distribution of utilization distributions under the hypothesis of independence) between kernel utilization distributions^40^ estimated using the same smoothing parameter determined above and a grid of 8,000 m. The pixels of the grid outside the home range over which the utilization distribution was estimated was set to zero. To compare overlap of seasonal home ranges and core areas, overlap indices were rescaled to the [0,1] interval by dividing them by the highest possible value (0.95 and 0.50, respectively)^41^.

### Habitat selection

 To assess the influence of environmental conditions (Supplementary Information, Table S2) on the spatial distribution of EGSB bowhead whales, we used a modified resource selection function^42^. We used the following broad-scale environmental covariates: water column depth (m), sea surface temperature (as a proxy for water mass; SST; ºC), a categorical variable for whether (or not) locations were inside the main ice edge, sea ice concentration, and sea ice concentration squared to account for preferences for intermediate ice concentrations. Prior to model fitting, each of the environmental covariates were transformed to improve model fits (Table S2). Notably, depth data were heavily bimodal (primarily using the continental shelf and to a lesser extent deep waters > 2000 m) and so a natural cubic spline was used to account for non-linear effects and facilitate interpretation of how topographical features were selected by bowhead whales (see Supplementary Information for additional details). All transformed covariates were then centered and scaled by subtracting the mean and then dividing by the standard deviation so that they had a mean of 0 and standard deviation of 1 to enable comparison of the relative influence of different environmental conditions on bowhead habitat use.

To estimate the habitat available to the tracked bowhead whales, we simulated a set of 100 track segments from each state-space model fit using the R package *aniMotum*^36^. Simulations were performed using the Mercator projection with the same starting coordinates as predicted track segments. Simulated track segments had the same number of locations (and time stamps) as predicted track segments, using the movement parameters from fitted models. Thus, they represented locations that could have been used by bowhead whales in the absence of habitat selection bias. Simulations were made using a gradient raster to include land as a barrier to whale movement. Due to the relatively unconstrained nature of the simulation, we used a similarity filter to retain 20 simulated track segments (those that were most similar based on displacement and bearing for each predicted track segment) to identify and remove less realistic simulated tracks^36,42,43^. Simulated track segments were then re-routed around land using the same criteria as predicted locations from state-space model fits (see above). Re-routing simulated tracks without the use of a gradient raster resulted in artificially inflated use of habitat immediately along the coast. Track segments were simulated for each model fitted to GW19-01 track segments, which were then truncated based on the same criteria as predicted locations. An inherent challenge associated with this methodology is that both the used and available locations are autocorrelated, which biases estimates of variance so that they are unrealistically small. To mitigate this issue, we used a modified resource selection function (described in^42,44,45^). We randomly assigned each of the 20 simulated track segments from the 86 state-space modelled track segments to a model dataset with its corresponding observed track segment. Each of these 20 model datasets were then fitted as 20 separate logistic regressions (Monte Carlo model fits) producing a set of 20 parameter estimates. The parameter estimates and their standard errors were taken to be the mean value of parameter estimates and their standard deviations across Monte Carlo model fits. To obtain *p*-values for each parameter, we tested the null hypothesis that regression coefficients were equal to zero using the means and standard errors from the 20 model fits. This assumed that parameter estimates were normally distributed which was visually confirmed. A likelihood-based model-selection procedure (e.g., Akaike Information Criterion) could not be used because the means and standard errors obtained for each logistic regression parameter were based on Monte Carlo methods. Model selection followed a backward stepwise procedure using *p*-values. The general form of our resource selection function models was an ordinary logistic regression with a logit link and binomial errors. The response variable *use* indicated whether the location was an available (simulated) location (0) or a used (predicted) location (1) and related it to a series of environmental covariates (*x*) by estimated parameters (β). The basic structure of the model was:

1 $$\:\mathrm{l}\mathrm{o}\mathrm{g}\mathrm{i}\mathrm{t}( \eta_{i,j})\:= \:\beta_0 + \:\beta_1\mathrm{x}_{1,i,j} +\beta_2\mathrm{x}_{2,i,j}\:+\:\dots\:\:+\:\beta_{\rm p}\mathrm{x}_{\mathrm{p},{i},{j}}\:+\:v_j$$

2 $$use_{ij} \sim {\rm Binomial}(\eta_{i,j} , 1)$$

where *i* indexes the predicted and simulated locations of the *j*th track segment and *v*_*j*_ is the random effect of the *j*th track segment (to account for individual identity). Whereby η_*i, j*_ is the linear predictor, which is logit linked to observations (*use*_*i, j*_) via a binomial error function^44^.

### Influence of environmental conditions on behaviour

To infer behaviour and make inferences about the movement patterns of EGSB bowhead whales, we fitted a move persistence model using joint estimation (i.e., a hierarchical state-space model which uses a single, pooled random variance parameter) to predicted locations (which were re-routed) using state-space model fits with the R package *aniMotum*^36,46^. This model uses time-varying move persistence (γ_t_) estimated using autocorrelation in both speed and direction as a behavioural index that varies continuously between 0 (low move persistence, commonly inferred to be apparent foraging) and 1 (high move persistence, commonly inferred to be traveling)^46–48^. After model fitting, track segments were combined by individual deployment (i.e., by whale and by year) into tracks. Track segments for GW19-01 were modelled separately, truncated as previously described, and then combined.

To assess the influence of environmental conditions on movement behaviour, we modelled logit-transformed γ_t_ (an output of the move persistence model) against transformed environmental covariates (Supplementary Information). These included static topographical features that influence oceanographic processes and enhance productivity (i.e., depth (m; modelled using a natural cubic spline to accommodate non-linear effects), slope (º), distance to the shelf break (km), distance to the ice edge (km), and distance to marine-terminating glacier fronts (km)) as well as more dynamic conditions (i.e., sea ice concentration, sea ice concentration squared to account for relationships with intermediate ice concentrations, SST (ºC), and sea surface height (m)) (see Supplementary Information). Barotropic streamfunction and sea surface height were collinear, so only sea surface height was included in the model. Given the small number of locations present in polynyas we did not include this feature as a predictor in tested models. We fitted linear-mixed effects models using the R package *nlme* version 3.1–168 with the following model structure:

3 $$\:\mathrm{l}\mathrm{o}\mathrm{g}\mathrm{i}\mathrm{t}(\gamma_{ \mathrm{t},j})\:=\:\beta_0\:+\beta_1{x}_{1,tj} + \beta_2 x_{2,tj} +\:\dots\:+\: \beta_{\rm p}\mathrm{x}_{\mathrm{p},t,j} + \phi \mathrm{l}\mathrm{o}\mathrm{g}\mathrm{i}\mathrm{t}(\gamma_{ \mathrm{t}-1,j})\:\rm V_\mathrm{j} + \varepsilon_{ \mathrm{t},\mathrm{j}}$$

where *x* values represent the covariates, β values are the respective estimated parameters, φ is the autocorrelation parameter, V_j_ is the individual random effect, and ε_t, j_ represents the uncorrelated residual variation^49^. We included individual identity as a random effect and used a first-order autoregressive structure (AR1) so that the bias in variance and parameter estimates due to autocorrelation were controlled. We fitted a series of candidate models (Table S3) using maximum likelihood estimation and selected the best-fitting model using AICc. Parameter estimates from the best-supported model were obtained using restricted maximum likelihood estimation. Normalized residuals were visually inspected to ensure model assumptions were met and variance inflation factors were used to check for collinearity. To assess performance of the best-supported model, leave-one-out cross validation was used where each individual included in the model was iteratively left out, the model was run at each iteration, and the coefficient estimates relative to the full model including all individuals were compared. This included the quantile range (5% and 95%) for the model including all individuals and the estimated trend for the percentage of cross-validation models that fell within the 95% confidence intervals for the model including all individuals^48^.

### Spatio-temporal patterns in move persistence

To explore individual variability in habitat use, we visually assessed spatio-temporal patterns in move persistence across individuals. We also inspected habitat use among individuals across months of the year. Bowhead whales are known to produce songs associated with reproduction from October to April in this population^50^. As such, the distribution of locations during these months could indicate breeding areas. We iteratively looked at each month to determine if there were obvious patterns in overlap among individuals and qualitatively assessed patterns.

## Results

A total of 85,270 locations (85,151 Argos, 119 Fastloc GPS) were collected from 40 instrument deployments on 38 bowhead whales (Table 1). For Argos locations, there were 54,421 class B, 14,334 class A, 9,612 class 0, 4,330 class 1, 1,700 class 2, and 754 class 3 locations. Argos error ellipse information was available for 84,526 Kalman-filtered locations, while the location classes for 625 locations were determined using least-squares. Tracking durations ranged between 2.9 and 632.1 days with a mean and standard deviation of 179.3 ± 136.3 days. There was a total of 5,273 tracking days (the number of days with at least one location) with a median of 147.5 days across deployments, ranging between four and 263 days with an average of 131.8 ± 91.8 days. The horizontal displacement speeds between consecutive locations were typically low with a median of 0.26 m s^− 1^ (mean and standard deviation of 0.35 ± 0.32 m s^− 1^). After filtering to remove implausible locations, 64,244 locations were fitted to continuous-time state-space models regularized at a four-hour time step, which resulted in a total of 27,768 locations across 39 tracks separated into 86 track segments from 37 individuals. Location data from individual GW18-02 were removed during filtering based on a short tracking duration and prevalence of large gaps in the data, which resulted in insufficient data to form track segments. After combining tracks for individual GW19-01 (which had tracking data from three instruments), a total of 27,710 locations were available for further analysis. Individuals instrumented in this study were all considered adults based on body size and colouration, except for GW21-01 which was thought to be a large, dependent calf. Most animals tagged were located within open water areas on the EGS, while three were located northwest of Svalbard in the polar drift ice (Fig. 2). Given the opportunistic nature of encountering bowhead whales in heavily ice-covered waters and the lack of obvious sexual dimorphism between sexes, only a small number of known females (based on genetic analysis) were equipped with tags in our study (3 females, 16 males, 19 unknown sex; Table S1).

### Home range

**Fig. 3:**
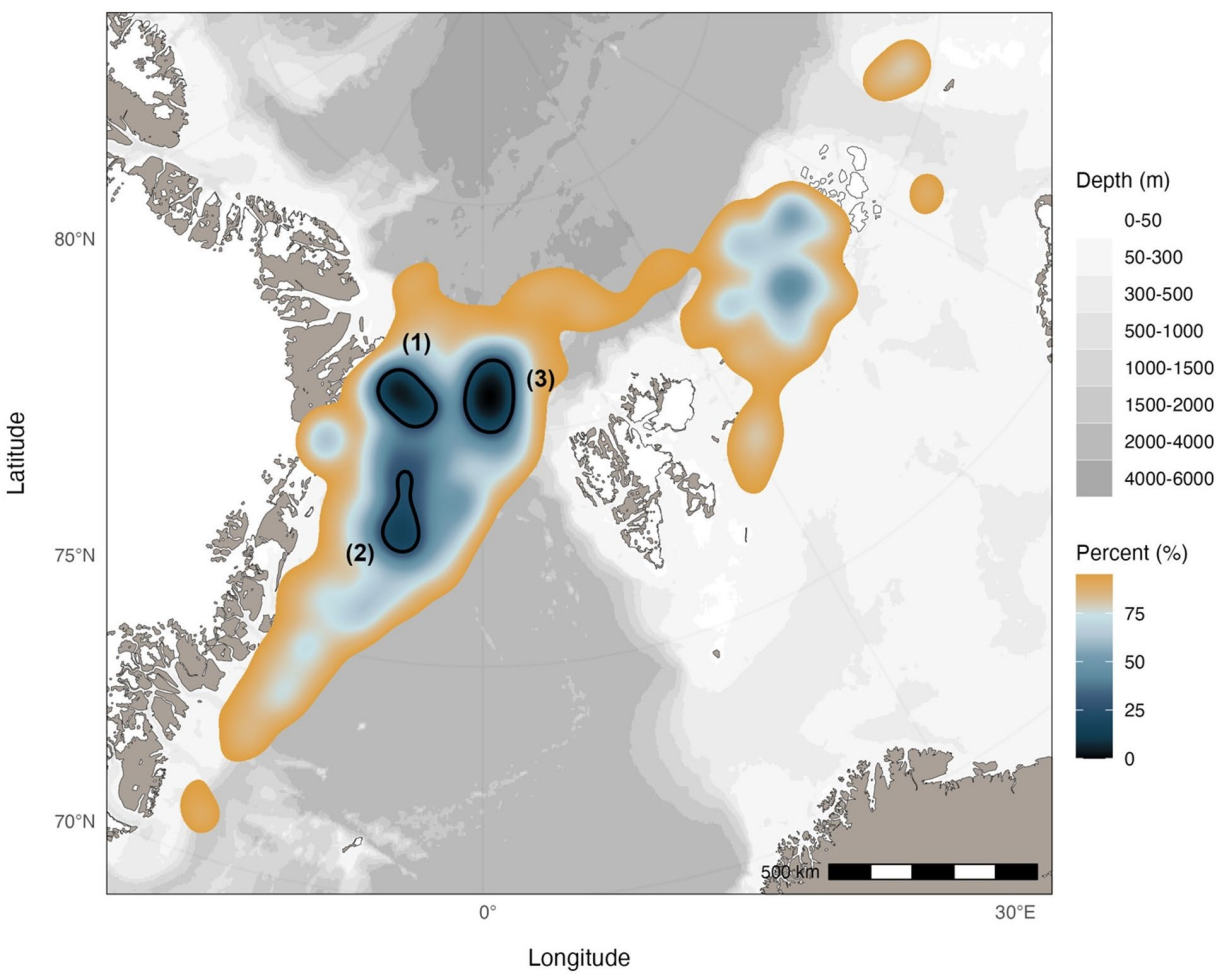
The home range (95% utilization distribution) of EGSB bowhead whales (*n* = 37). The utilization distribution was estimated using kernel density estimation and is represented as a gradient within the home range. The 25% utilization distributions (hotspots) are included with black lines and labelled (1), (2), and (3). The map was produced using the R package *ggOceanMaps*^33^.

 The home range (95% utilization distribution) of EGSB bowhead whales spanned from the EGS across a narrow corridor above Svalbard and over to FJL and constituted 666,449 km^2^ (Fig. 3). Although a small number of locations occurred near Svalbard (Fig. 2), the home range did not extend to coastal areas of the archipelago (Fig. 3). Two core areas (50% utilization distribution) occurred over the northern part of the EGS (107,569 km^2^) and in waters surrounding western FJL (2,943 km^2^). There were three hotspots (25% utilization distribution) located at (1) the northeastern tip of Greenland (12,871 km^2^), (2) over the outer edge of the EGS (9,791 km^2^), and (3) the largest, extending over offshore (> 200 km), deep (> 4,000 m) waters of the central Fram Strait and over the northwestern outer edge of the shallower Yermack Plateau (500 to 1,000 m) (15,846 km^2^) (Fig. 3). The number of predicted locations available for home range analyses were highest in fall following instrument deployment, however the number of locations available were generally high throughout the year (Table S4). Individuals typically dispersed from the location of instrument deployment, reducing but not eliminating potential bias of deployment location on home range estimation.

**Fig. 4:**
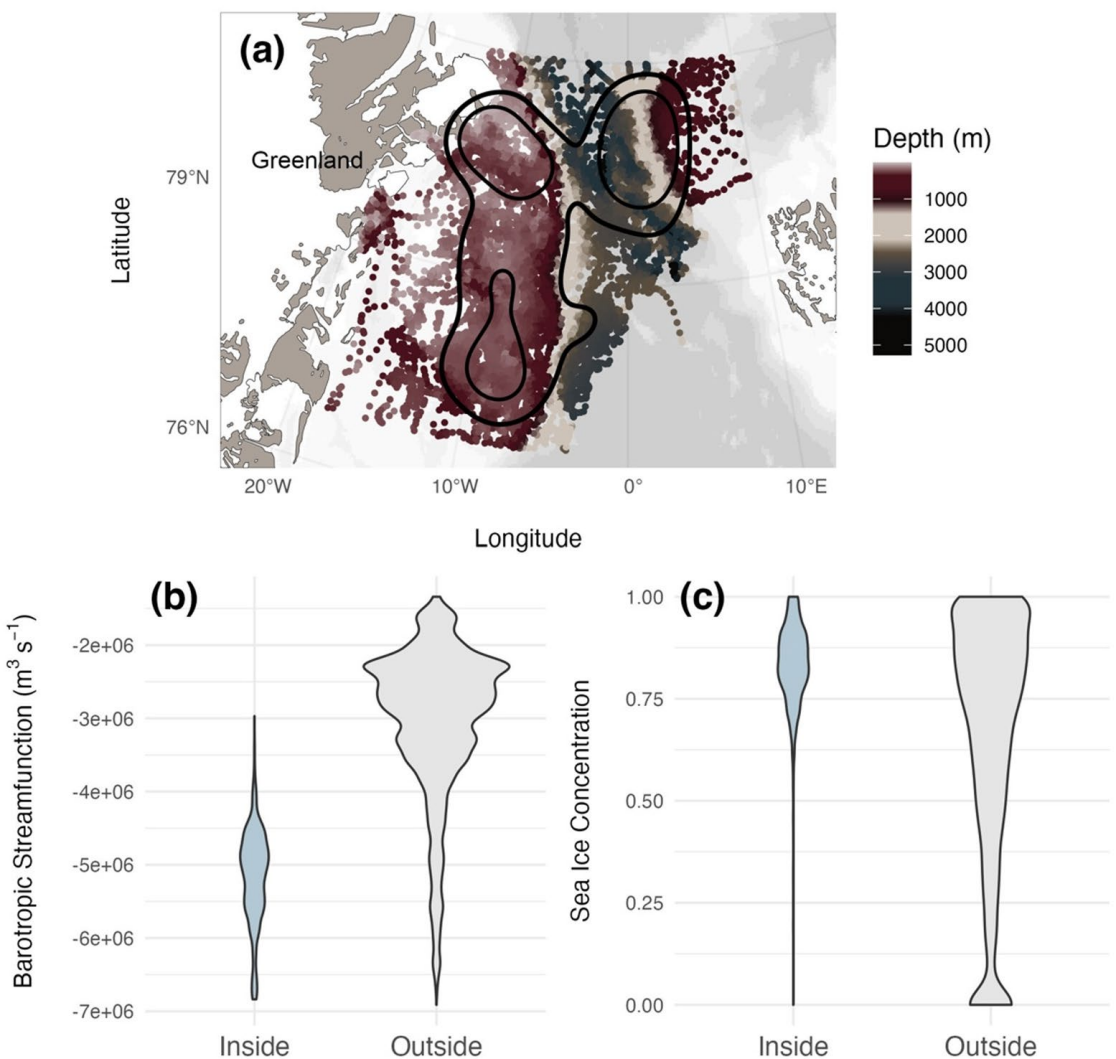
Environmental conditions within the 50% (core areas) and 25% (hotspots) kernel utilization distributions (KUD) for EGSB bowhead whales. (**a**) Spatial distribution of bowhead whale locations within the core area (50% KUD) and hotspots (25% KUD) coloured by water column depth (m) with outlines of core area and hotspots in black and violin plots for the (**b**) barotropic streamfunction (m^3^ s^− 1^) and (**c**) sea ice concentration at locations inside the largest hotspot (Fig. 3 hotspot 3) and all locations outside of it. The map was produced using the R package *ggOceanMaps*^33^.

Although the hotspots identified appear to have defined boundaries, the distribution of locations within these hotspots suggest that fine-scale features may be important in structuring the distribution of bowhead whales within them (Fig. 4a). The largest hotspot (Fig. 3 hotspot 3; over deep water and edge of the Yermack Plateau) was used 11 months of the year (all months except September) and nearly all of the tagged individuals used this hotspot at some point in time. In this offshore, deep-water hotspot, water depth (W = 13656551, *p*-value < 0.001), the slope of the sea floor (W = 81949906, *p*-value < 0.001), the absolute value of cyclonic barotropic streamfunction (W = 4495642, *p*-value < 0.001), and the deviation in sea surface height above geoid (W = 11845427, *p*-value < 0.001) were all significantly greater compared to locations in the rest of their habitat (inclusive of other hotspots) (Fig. 4). The depth at locations inside the largest hotspot ranged from 698 m to 4,340 m deep, with a median depth of 2,291 m (mean and standard deviation of 2,146 ± 763 m). The median sea ice concentration of locations within this hotspot was 84% and of the 4,107 locations within it, only five were located in open water outside the ice edge. Through visual assessment, the eddy kinetic energy appeared to be substantially higher and more dynamic in this region (and the area immediately south of it) across all seasons (Fig. S1) compared to other areas within the distributional range of EGSB bowhead whales.

 Individuals inhabited the northernmost and southernmost parts of their range in both summer and winter (Figs. S2 and S3). The seasonal population home ranges were not markedly different and the overlaps in utilization distributions were generally high between seasons (Table S5), with consistent use of both core areas (the EGS and FJL) across all seasons (Fig. S4). The seasonal core areas used differed to a greater degree and while the same general areas were used, overlaps between utilization distributions were low (Table S5). Instrumented whales spent more time close to the shelf break and ice edge in summer, whereas fall movements were more concentrated over the continental shelf. Individuals tended to avoid coastal areas (associated with formation of fast ice) in winter and spring. The use of the offshore, deep-water hotspot persisted across all seasons.

### Habitat selection

**Fig. 5:**
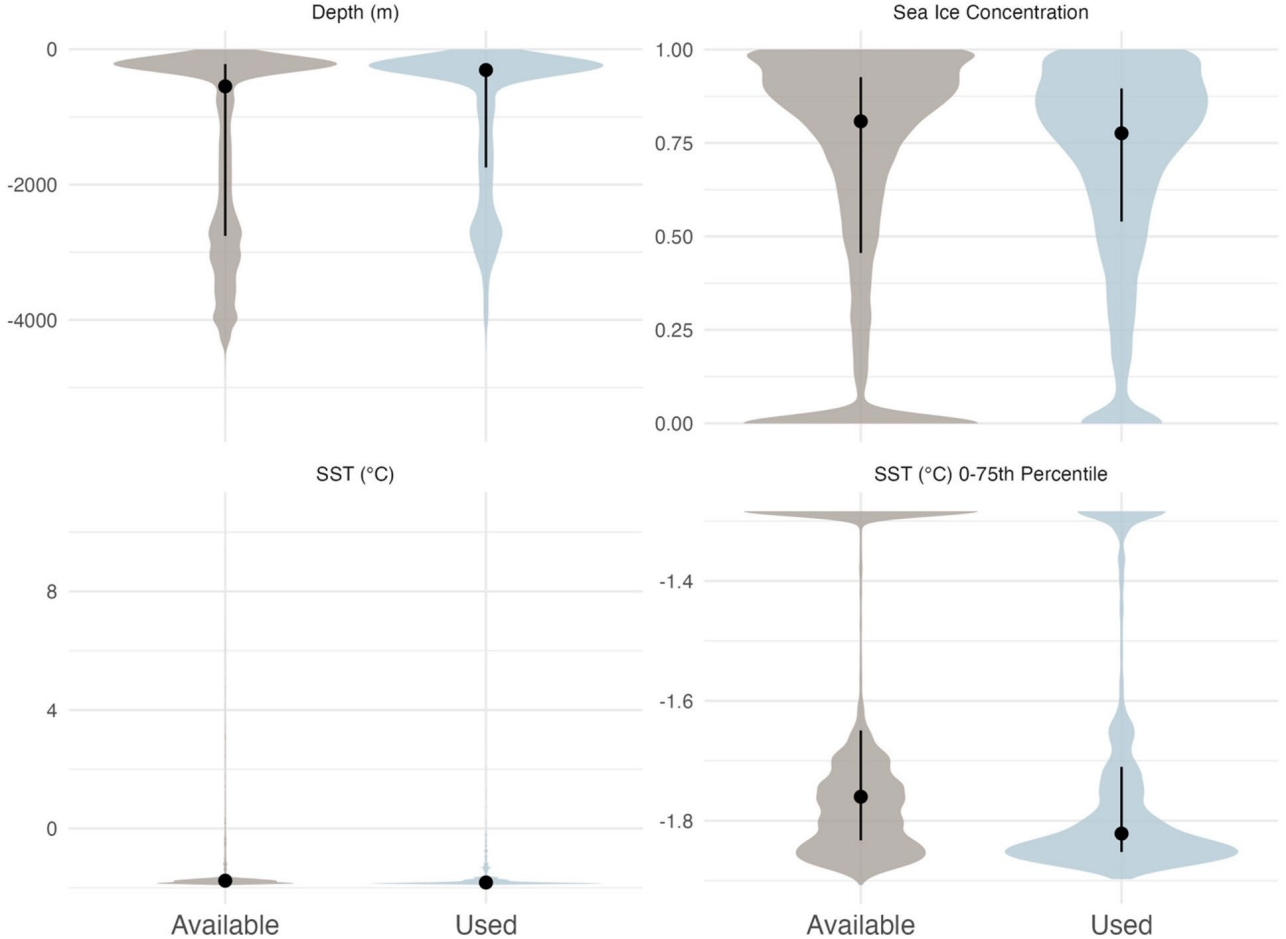
Violin plots of environmental covariates included in the modified resource selection function at available and used locations by EGSB bowhead whales. Environmental conditions include depth (m), sea ice concentration, and sea surface temperature (SST; ºC) as well as the 0 to 75th percentile of SST to highlight the spread of the data.

Broad-scale environmental features were important for the habitat selection of EGSB bowhead whales (Table 2; Fig. 5). SST was the most important single linear predictor of bowhead whale habitat use. Bowhead whales were less likely to use areas with higher SSTs (i.e., Atlantic Water) and showed a strong preference for Arctic Water (mean and standard deviation of −1.60 ± 0.62 °C for used locations) (Figs. S5 and S6 for distributions of used and available locations and SSTs).

**Table 2:** Results of the modified resource selection function for habitat use by EGSB bowhead whales including the mean coefficient estimate, lower and upper 95% confidence intervals (CI), and *p*-values calculated for each environmental covariate. The covariate names include depth as depth (m) modelled with a natural cubic spline (knots at 220 m, 500 m, 2500 m), sst as sea surface temperature (ºC), in_ice_ as a categorical variable whether (or not) locations were inside the main ice edge, ice as sea ice concentration, ice_sq_ as sea ice concentration squared.

Covariate	Lower 95% CI	Coefficient Estimate	Upper 95% CI	*p*-value
dpth_1_	−0.137	−0.081	−0.024	**< 0.01**
dpth_2_	0.394	0.444	0.494	**< 0.001**
dpth_3_	−0.591	−0.491	−0.392	**< 0.001**
dpth_4_	−2.100	−2.005	−1.910	**< 0.001**
sst	−1.073	−1.025	−0.978	**< 0.001**
in_ice_	0.628	0.692	0.756	**< 0.001**
ice	−0.600	−0.534	−0.469	**< 0.001**
ice_sq_	−0.264	−0.239	−0.214	**< 0.001**

Locations being inside the main ice edge was the second most important single linear predictor of habitat use. Almost 90% of locations (24,827 out of 27,710) were inside the main ice edge and most locations outside the main ice edge were relatively close to the edge (median of 30.2 km and mean and standard deviation of 63.2 ± 71.4 km). Locations were inside the ice edge most frequently in spring (only 0.98% of locations outside the ice edge, compared to 1.5% in fall, 28.1% in winter, and 14.7% in summer). Locations inside the main ice edge were also the farthest inside the ice edge (189.5 km and 212.2 ± 111.2 km) and locations outside the main ice edge (in open water) were closest to it during spring (18.61 km and 17.2 ± 11.7 km).

Across bowhead whale habitat, depth was an important non-linear predictor of EGSB bowhead whale habitat use, with significant and variable effects depending on topographical features (Fig. S7). The depths used by whales was also heavily bimodal (Fig. 5). Nearly half of all locations occurred over the continental shelf in depths ≤ 300 m (48.70%). When including deeper parts of the continental shelf (up to the shelf break at 500 m depth) these depths accounted for most locations (61.7%). A second peak in use occurred between 2,000 m and 3,000 m (15.93%).

Selection increased toward deeper parts of the continental shelf (possibly due to the presence of fast ice in some seasons) and peaked at the transition to deeper continental shelf features. Bowhead whales showed a marked preference and the strongest selection for depths between 220 m and 500 m (i.e., gullies and troughs or the continental shelf slope). Together with the disproportionate use of this depth range, these results indicate that the continental shelf is particularly important habitat, which is consistent with their widespread distribution over the EGS (Figs. 2 and 3), which averages approximately 200 m depth. Habitat selection was significantly lower and declined rapidly past the continental shelf break (500 m to 2500 m). Deep water (> 2500 m) was most strongly avoided, suggesting these regions (i.e., Nansen or Amundsen Basins) are not suitable habitat for bowhead whales. While depths of around 2,500 m (i.e., the offshore hotspot and travel corridor) were used, these depths were still avoided relative to their availability.

Although the whales clearly preferred being inside the ice edge, as sea ice concentrations increased towards 100%, use by bowhead whales decreased. The inclusion of a squared sea ice concentration was significant, suggesting that as sea ice concentrations increased, the effect of sea ice concentration on use decreases. At lower sea ice concentrations, habitat use was more likely to be in open water areas and once they were in relatively high sea ice concentrations, the precise sea ice concentration was not as important. Despite this relationship, sea ice concentrations used by bowhead whales were generally high with a median of 77.6% (mean and standard deviation of 67.9 ± 28.9%). Median sea ice concentrations used by bowhead whales were highest in winter (86.8%) and spring (87.6%) compared to summer (44.0%) and fall (68.8%). Given almost all locations were present inside the main ice edge, open water (ponds within ice fields) and areas with very low sea ice concentrations inside the ice edge appear to be important bowhead whale habitat.

### Influence of environmental conditions on behaviour

**Fig. 6:**
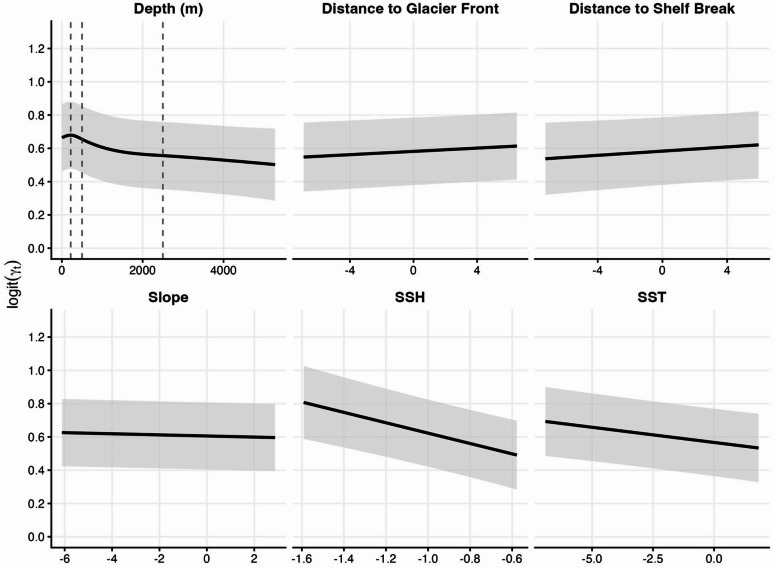
Partial response curves for environmental covariates of the best-supported linear mixed-effects model for logit-transformed move persistence (γ_t_) of EGSB bowhead whales. Environmental covariates were transformed and include depth (m) modelled as a natural cubic spline with knots at 220 m, 500 m, and 2500 m (dashed lines), distance to glacier front, distance to shelf break, slope, sea surface height (SSH), and sea surface temperature (SST). Values were calculated by varying environmental covariates while holding other predictors at their mean and 95% confidence intervals are included as grey bands.

The results of the linear mixed-effects model suggest that low move persistence was associated with specific environmental conditions encountered by EGSB bowhead whales (Fig. 6). The best-supported model for move persistence included water depth, slope, distance to the shelf break, distance to marine-terminating glacier front, SST, and sea surface height (Table 3; Figs. 6 and S8). Considering the number of predictors included in our model and the complexity of regional oceanographic processes, we did not compare the relative importance of the different environmental conditions. Nevertheless, depth was an important predictor of bowhead whale move persistence and it was included in the best 141 candidate models, followed by sea surface height in the best 120, SST in the best 61, and distance to marine-terminating glacier front in the best 21 (Table S3), suggesting the most important predictor for apparent foraging behaviour was depth (a static feature which structures zooplankton distributions both in space and by species), followed by dynamic features associated with water masses and circulation patterns in this region, and then glacier fronts (which promote both upwelling and primary productivity).

**Table 3:** Coefficient estimates, standard errors, and *p*-values of environmental covariates for the best-supported candidate linear mixed-effects model for logit-transformed move persistence (γ_t_) of EGSB bowhead whales (Table S3). Coefficients with significant *p*-values are indicated in bold. The quantile range (5% and 95%) of parameter estimates from leave-one-out cross validation of the most parsimonious model are presented with the estimated trend (Est. Trend), which represents the percentage of cross validation models where the estimated coefficients fall within the 95% confidence intervals of the parameter estimates from the model including all individuals. The covariate names include depth as depth (m) modelled with a natural cubic spline (knots at 220 m, 500 m, and 2500 m), slp (º) as sea floor slope, dst_shlf_ as distance to the shelf break (500 m isobath) (km), dst_glc_ as distance to the nearest marine-terminating glacier front, sst as sea surface temperature (ºC), and ssh as sea surface height above geoid (m).

Covariate	Coefficient Estimate	Standard Error	*p*-value	Leave-One-Out Cross Validation		
				Lower	Upper	Est. Trend
Intercept	0.273	0.124	**< 0.05**	0.030	0.516	100.0
dpth_1_	−0.090	0.027	**< 0.01**	−0.142	−0.038	97.3
dpth_2_	−0.115	0.035	**< 0.01**	−0.183	−0.047	100.0
dpth_3_	−0.121	0.039	**< 0.01**	−0.198	−0.045	100.0
dpth_4_	−0.175	0.046	**< 0.001**	−0.265	−0.085	97.3
slp	−0.003	0.002	0.057	−0.007	0.000	100.0
dst_shlf_	0.006	0.004	0.087	−0.001	0.014	100.0
dst_glc_	0.005	0.002	**< 0.05**	0.001	0.009	100.0
sst	−0.018	0.005	**< 0.001**	−0.029	−0.008	100.0
ssh	−0.312	0.068	**< 0.001**	−0.445	−0.178	94.6

Depth was a primary driver of move persistence in EGSB bowhead whales, with statistically significant variation associated with topographical features (Fig. 6). Move persistence was highest in shallow continental shelf waters (0 m to 220 m), with a modest local maximum of move persistence (inferred as travel) at 220 m, then sharply decreasing (inferred as increasing apparent foraging) across deeper parts of the shelf and slope (220 m to 500 m), and continued to decline more steadily over deep offshore waters (500 m to 2500 m), with the lowest move persistence associated with the greatest depths (> 2500 m) used by the whales (Fig. 6). The first derivative reached its minimum (maximum negative rate of change) at 500 m, indicating bowhead whales most rapidly changed behaviour from travel to apparent foraging immediately once they passed the shelf break (Fig. S9).

Although it was not a significant predictor, the inclusion of slope improved model fit, and as slope increased individuals were more likely to demonstrate apparent foraging behaviour. Similarly, although distance to the shelf break was not a significant predictor, it was retained in the best-supported model. As distance to the shelf break increased, the whales were more likely to be traveling, indicating that residency behaviour occurred closer to the shelf break.

Distance to marine-terminating glacier fronts was a significant predictor of move persistence. Individuals were more likely to have lower move persistence or demonstrate residency behaviour closer to glacier fronts. As SSTs used by the bowhead whales increased, individuals were more likely to have lower move persistence. However, SSTs used by the whales occurred within a narrow range (−1.90 °C to 4.30 °C) and were generally very low (median of −1.82). As the deviation in sea surface height increased, individuals were more likely to have lower move persistence. Residual diagnostics indicated model assumptions were met and variance inflation factors confirmed multicollinearity was not present between predictor variables. Leave-one-out cross-validation demonstrated that the best-supported model performed well across all iterations (Table 3).

### Spatio-temporal patterns in move persistence

**Fig. 7:**
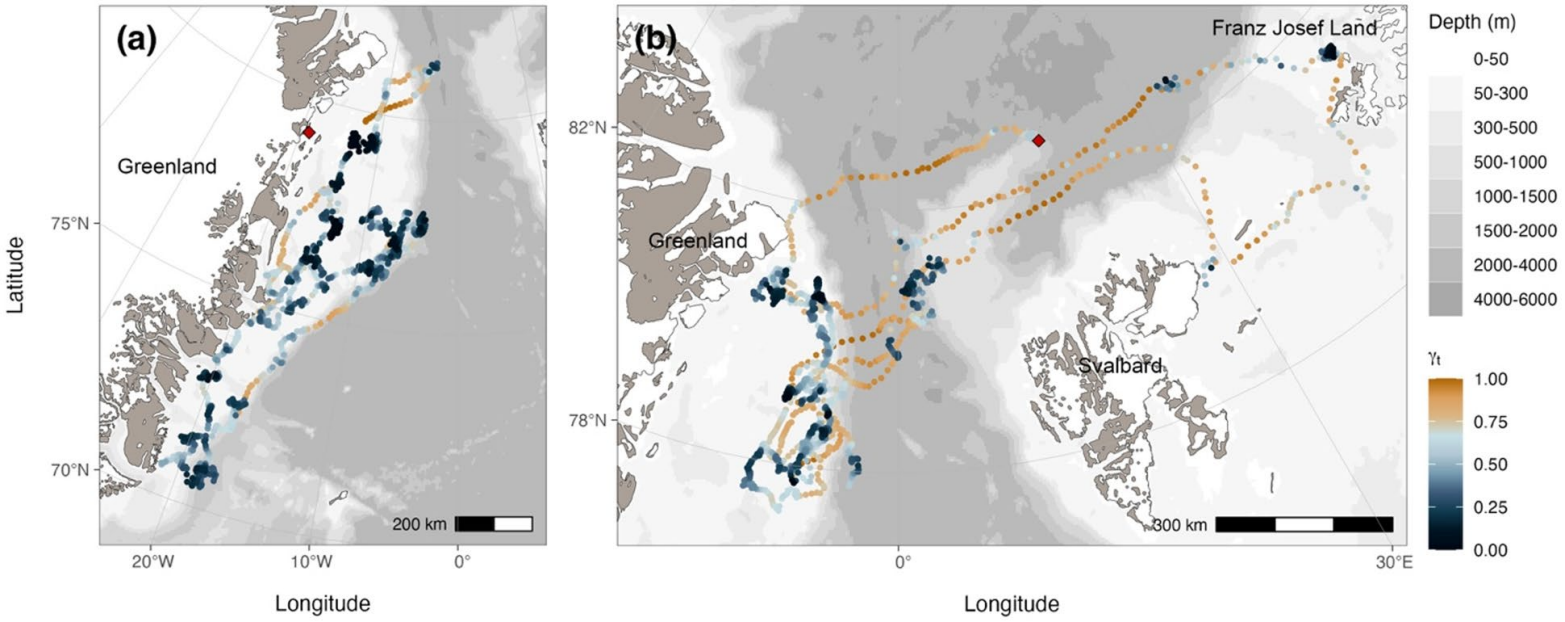
Move persistence along tracks for two male EGSB bowhead whales. Locations are shown for (**a**) GW21-05 and (**b**) GW21-12 which were instrumented summer/early fall of 2021 and spring of 2022, respectively, and are coloured by normalized move persistence estimates (γ_t_). Instrument deployment locations are included as red diamonds. Maps were produced using the R package *ggOceanMaps*^33^.

**Fig. 8:**
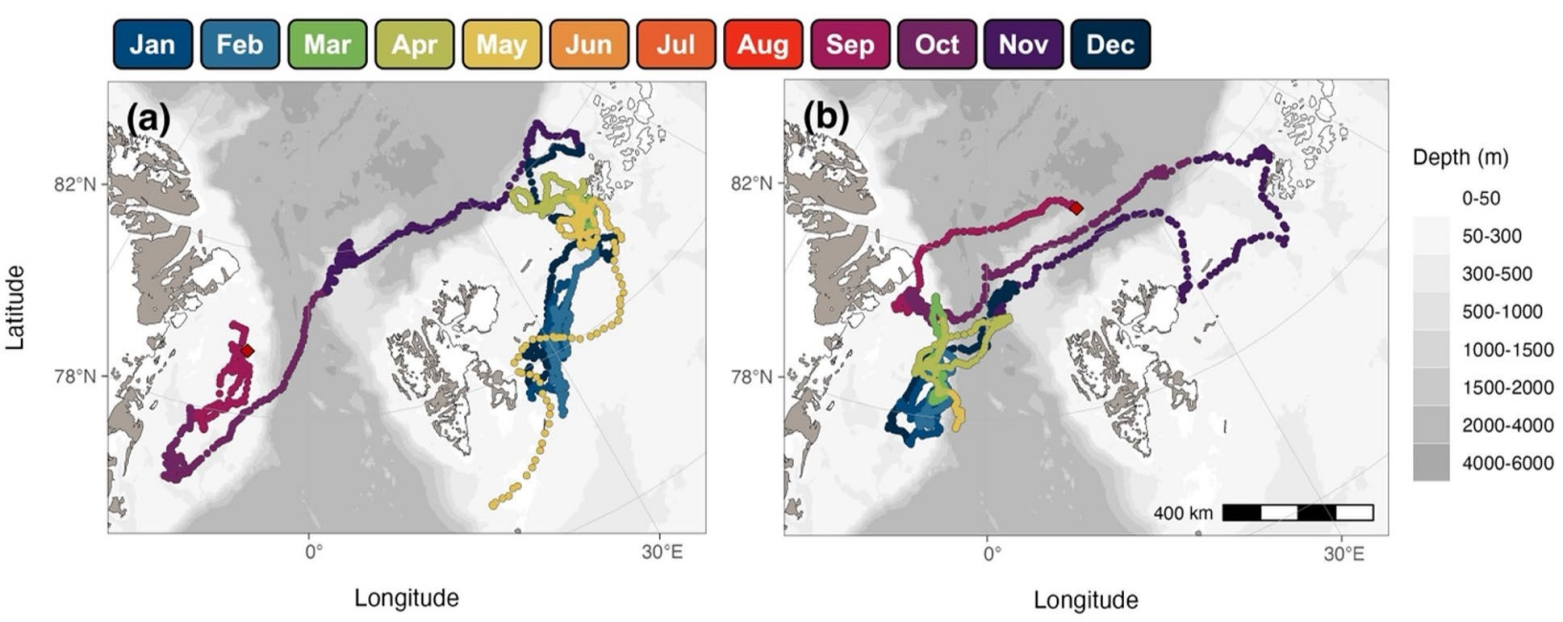
Seasonal movement patterns of two male EGSB bowhead whales. Locations are shown for (**a**) GW20-02 and (**b**) GW21-12 which were instrumented between September and May and are coloured by month. Instrument deployment locations are indicated by red diamonds. Maps were produced using the R package *ggOceanMaps*^33^.

Spatio-temporal distributions and movement patterns were not consistent among individuals instrumented in our study (Figs. 2 and S2). For example, two individuals that were instrumented in the same deployment period (late summer/early fall 2021 to spring 2022) exhibited striking differences (Fig. 7), one remained on the EGS while the other performed multiple movements back and forth between the EGS and FJL. Notably, travel between the EGS and FJL was performed by only nine instrumented individuals while most others remained on the EGS. Individuals that performed long-distance trips between these regions did not do so on a classical seasonal basis (Fig. 8). Although movements from the EGS to FJL appeared to most commonly occur in fall (concurrent with the minimum extent of sea ice), this was not always the case and even when these movements did occur in fall, winter was not always spent in the Barents Sea (Fig. 8). Individuals also travelled between these areas in winter, spring, and summer (Figs. S2 and S3a). The specific timing and direction of movements was also not consistent, with some spending winter in FJL and others spending summer there (Figs. 8 and S3a). For the whales that remained on the EGS, no obvious seasonal patterns in northward or southward movements were exhibited. Although there was presumably some influence of the extent of sea ice, some individuals travelled south in winter (Fig. S3b) and some travelled north in winter (Fig. S3c), but many did not exhibit much seasonal structure in their distribution at all (Fig. S3d).

By visually comparing the spatial distribution of individuals over months when singing is known to occur, which is assumed to accompany mating, we determined that overlap was most pronounced in the months November to February. Of 22 individuals that had location data during this period, 20 were located within a relatively small area spanning the outer edge of the EGS and in the Fram Strait (offshore area) during at least part of this period, corresponding to areas of residency behaviour and hotspots 2 and 3 (Figs. 2 and 3). Movements between these hotspots did not have set temporal patterns (i.e., individuals occupied both areas and moved between them in all months). Although there was only one female with data available during this period, her movements were consistent with those of males and individuals of unknown sex.

## Discussion

The distribution and movement patterns of EGSB bowhead whales appear to be closely associated with the environmental conditions that structure their Arctic habitat. Habitat use was associated with low SSTs and being inside the ice edge but was limited by deeper water depths over the Fram Strait and northern parts of their range, likely due to a lack of prey availability in these areas. Movement patterns, and presumably foraging behaviour, were strongly influenced by dynamic oceanographic conditions (sea surface temperature and height) at the interface between Atlantic Water and Arctic Water, an area disproportionately affected by climate change, but static features (water column depth and proximity to marine-terminating glacier fronts) were also important. A large core area offshore over deep (> 4000 m) water was occupied year-round, which likely serves as both a foraging area and a breeding ground based on residency both during and outside the mating season. The reliance of EGSB bowhead whales on this type of habitat is unique compared to other populations of bowhead whales and contributes to their vulnerability given projected changes in regional hydrography and sea ice conditions^51^. The patchy distribution of apparent foraging behaviour of bowhead whales also sheds light on the spatio-temporal distribution of secondary productivity in this ecosystem, which are presumably high enough to support the foraging requirements of an exceptionally large marine predator throughout the year, including the polar night.

The effects of Atlantification and Arctic Amplification (faster warming than the global average due to albedo and other feedback mechanisms) are enhancing primary production in the Arctic^52^. While these changes are likely improving the current foraging conditions for EGSB bowhead whales, continued warming and concomitant declines in sea ice are expected to reduce suitable habitat and the abundance of lipid-rich traditional prey populations for this habitat specialist^53^. K-selected life histories, exemplified by the bowhead whale, can limit the capacity and speed at which populations can adapt to environmental change. In such circumstances, other coping strategies become more important^8^. Although range expansion may not be possible given the environmental characteristics associated with preferred habitat and apparent foraging, intrapopulation variability in movement patterns (and presumably foraging behaviour) suggests a degree of plasticity at the population scale that may buffer some of the negative effects of climate change. The results of our study highlight the importance of assessing vulnerability to climate change across a species’ range to identify intrinsic and extrinsic sources of vulnerability and serves as a useful example for understanding the resilience strategies available to at least some habitat specialist species. The statistical challenges and limitations of our study are described in the Supplementary Information.

### Habitat specialisation

The EGSB population of bowhead whales exemplifies the qualities of an Arctic specialist species. They nearly exclusively inhabit cold Arctic Water (−1.9 to 0 °C) inside the main ice edge and largely avoid warmer Modified Atlantic Water (0 to 2 °C) or Atlantic Water (> 2 °C) (Fig. S6 for distribution of SSTs at used and available locations). A strong preference for low SSTs is ubiquitous across bowhead whale populations, as well as other Arctic specialist cetaceans^45,51,54–56^. For example, in the BCB population, individuals are only recorded in the Chukchi Sea when temperatures fall below 2 °C^57^.

Previous research has suggested that the strong preferences for cold water among Arctic whales may be to avoid thermal stress^54^. Bowhead whales have the thickest blubber layer of any cetacean and while it supports thermoregulation (in addition to other functions) in cold water, it is primarily composed of unsaturated fatty acids which have low melting temperatures, posing a physiological limitation in warmer waters^10^.

The presence of an intraoral corpus cavernosum maxillaris, which allows bowhead whales to effectively dump excess heat to avoid the brain overheating, also supports the hypothesis that physiological limitations may be driving habitat use. When found in warmer waters (> 4 °C), ECWG bowhead whales have been observed molting and in some instances, swimming with open mouths^58^. The OKS population exhibits increased molting behaviour compared to other populations and also experiences substantially higher SSTs within its range (11.5 to 16.5 °C), which is believed to be contributing to this population declining^55,59^. Although it is possible that EGSB bowhead whales could also use warmer waters to facilitate molting, less than half of instrumented animals (*n* = 16) encountered SSTs above 0 °C and the spatio-temporal distribution was variable. Given the vertical distribution of warmer Atlantic Water in this region, analysis of temperature at-depth may provide more insight into the moulting behaviour of this population, but this topic is beyond the scope of this study. Thermoregulatory adaptations for colder environments (or narrow thermal ranges) can prove maladaptive as global temperatures increase^60^. These adaptations can become a source of vulnerability in species unable to respond to changing climates through range shifting (e.g., habitat specialisation, limited dispersal capacity, high latitude species).

The EGSB bowhead whale population demonstrates year-round affiliation with sea ice, spending most of their time inside the ice edge. The highest proportion of locations found outside the ice edge occurred in winter. Although killer whales primarily use echolocation to locate prey^61^, limited visibility during the polar night may make bowhead whales more inconspicuous to predators and increase willingness to move into open water. While an affinity for sea ice is characteristic of other bowhead whale populations, the relationships are somewhat different. For both the BCB and ECWG populations, movements typically follow the seasonal pattern of sea ice formation and recession^45,56,62^ and associations often occur at the ice edge where increased productivity promotes the aggregation of prey^56,63^. While some individuals did occupy habitat close to the ice edge, there was no clear trend (median distance of 132.39 km, ranging from 0.00 to 654.85 km). Furthermore, proximity to the ice edge and sea ice concentration were not important predictors of apparent foraging behaviour for EGSB bowhead whales. Our results suggest that sea ice use may not be specifically associated with prey availability, as seen in other populations. This is consistent with the spatial distribution of apparent foraging (Fig. 2) as well as the habitat characteristics of preferred *Calanus* zooplankton species in the region^29^.

The affiliation of bowheads with sea ice is likely genetically encoded as a means of natural predator avoidance, as it presents a barrier to movement to killer whales (*Orcinus orca*), their primary predator, which possess a large dorsal fin. The influence of predation risk or landscapes/seascapes of fear on habitat use and behaviour has been widely documented across both terrestrial^64^ and marine ecosystems^65^. In the Chukchi Sea, killer whales overlap with bowhead whales in summer (with evidence of predation) and are often found at the ice edge in winter^66^. In the eastern Canadian Arctic, the presence of killer whales has been shown to significantly influence foraging behaviour in bowhead whales. Bowhead whales shifted from use of open water in the absence of killer whales to dense sea ice and shorelines when they were present^67^. While predator avoidance provides the most plausible explanation for the affiliation of EGSB bowheads with sea ice, the extreme affiliation with sea ice seen in this population might be a response to historical whaling pressure in open water areas, leaving only those individuals that spent most of their time inside the ice edge^68^. The individual variability in movement observed in the present study supports the idea that multiple movement strategies exist in this population, which could have included some with greater use of open water prior to historical whaling. Currently, their ice-affiliation also limits habitat overlap with seasonally-resident zooplanktivorous mysticetes, which might exert further positive selection pressure^69^. As sea ice declines within their habitat, EGSB bowhead whales will likely need to modify their behaviour to balance increased predation risk and competition with reduced foraging opportunities^70^. Similar trade-offs have been observed in other cetaceans preyed upon by killer whales such as Cuvier’s (*Ziphius cavirostris*) and Blainville’s (*Mesoplodon densirostris*) beaked whales, which exhibit altered movement patterns as a risk-sensitive foraging strategy to reduce predation risk at the cost of foraging efficiency^71^.

Although bowhead whales in our study largely remained inside the sea ice edge, preference for relatively low sea ice concentrations and the fact that sea ice concentration was not an important predictor of behaviour suggest that open water areas within the sea ice are important habitat. If suitable foraging habitat can be found within (or near) ponds and leads, it is reasonable to assume bowhead whales should use these features to reduce the energetic costs of movement (i.e., breaking through dense sea ice or searching for open areas) once predator avoidance has been achieved. As ram filter feeders requiring high densities of small-bodied prey to meet their energetic needs, individuals should exploit as many prey patches as possible during movement (for example, both apparent foraging and traveling along the shelf break seen in Fig. 7).

At high sea ice concentrations, the relative sea ice concentration was not as important for habitat selection of EGSB bowhead whales. This could be due to ease of movement but also potential for encountering productive waters. Open water areas within the sea ice promote high concentrations of phytoplankton due to the subduction of surface meltwater and formation of submesoscale subsurface fronts^72^. These areas are also associated with under-ice blooms which have, until recently, been substantially overlooked in estimates of net primary production for the Arctic^52^. Increased absorption of solar radiation in these open water areas (i.e., decreased albedo) may also partially explain why slightly warmer SSTs were associated with apparent foraging^73^. Sea ice declines have resulted in increases in secondary production in other parts of the Arctic^74^ and have been associated with improved body condition in BCB bowhead whales^75^.

We were unable to assess whether apparent foraging by bowhead whales in our study was associated with polynyas because too few locations were classified as being in such areas. We suspect that this may be due in part to the resolution of available sea ice data as these areas are generally believed to be important for marine mammals in the Arctic, including bowhead whales^76–78^. The lack of polynya use might also be related to the dynamic nature of the sea ice along the coast of Greenland, with Arctic drift ice fields and large glacier pieces constantly flowing southward.

Continued warming of the Arctic will likely reduce suitable habitat for EGSB bowhead whales^51^. However, current increases in productivity may enhance foraging opportunities available to EGSB bowhead whales, without sacrificing protection from predation and competition in the region^79^. Apparent foraging behaviour (inferred from low move persistence values) was associated with slightly warmer SSTs. In some areas, foraging behaviour by the BCB bowhead whale population is also associated with warmer shelf waters^66^. However, the opposite trend is found in the Chukchi Sea, where lingering behaviour has been associated with colder surface waters^45^. These differences have been attributed to regional distributions of preferred prey (i.e., krill and larger calanoid copepods). These differences in prey distributions highlights the importance of considering inter- and intrapopulation variability in associations with environmental conditions as sources of extrinsic vulnerability.

Similar to many other Arctic marine mammal species^80^, marine-terminating glacier fronts provided important foraging habitat for EGSB bowhead whales. At local spatial scales (< 10 km), glacial meltwater and plumes of subglacial discharge aggregate zooplankton and directly impact vertical nutrient fluxes resulting in increased primary production. Mesoscale processes (spanning 10 to 100 km) can also play a significant ecological role by driving phenomena including nutrient upwelling, iron enrichment, changes to the carbonate system, and enhanced stratification^81^. These effects may be more pronounced in areas such as fjords or areas where there are many tide-water glacier fronts such as FJL (Fig. 2)^82^, which was used by approximately one quarter of bowhead whales instrumented in our study (Fig. 2). The use of FJL by only a subset of tagged whales suggests some level of resource partitioning occurs within this population, which may serve to reduce intraspecific competition, particularly when prey resources are less abundant. This population-level behavioural plasticity may foster resilience to the consequences of ongoing and projected climate change across their range. Reduction of this type of foraging habitat in both FJL and the east coast of Greenland is cause for concern^82,83^.

### Association with environmental conditions

The complexity of Arctic biogeography poses significant challenges in understanding the interactions among environmental drivers and their relationships with primary productivity dynamics^52^, as well as identifying the diverse conditions that provide suitable foraging habitat for upper-trophic level predators. Similar to other populations, EGSB bowhead whales predominantly used the continental shelf (EGS and Barents Shelf), which provides important foraging habitat for this species across its range^66,84^. Habitat selection was highest at deeper parts of the continental shelf, consistent with trends in zooplankton abundances in this region^85^. Bowhead whales may also be utilizing deeper troughs present on the continental shelf, which lead to exchanges between the shelf break and the EGS, including incoming nutrient-rich Atlantic Water^86^. This is consistent with increased apparent foraging behaviour in areas with greater slope angles. Although travel was most associated with being on the continental shelf, this was unsurprising given the strong preference for use over the continental shelf and limited use of deeper depths. Aside from travel to and from FJL, when animals did use deeper depths, movements were primarily associated with residency behaviour.

The importance of the shelf break as suitable foraging habitat for bowhead whales may be underrepresented in our study. Some individuals displayed both apparent foraging and travel behaviour when moving along the shelf break (e.g., Fig. 7), which is supported by strong preference for using deeper parts of the continental shelf. Conceivably, bowhead whales may travel along these more productive features to allow for opportunistic foraging when resources are present along their path (i.e., increasing the chances of encountering productive areas). This sort of movement pattern was not performed by all individuals, providing further support that there are multiple foraging strategies that enable bowhead whales to persist within this ecosystem.

### Offshore, deep-water habitat

We identified a hotspot for EGSB bowhead whales located offshore, partially extending over the deepest part of the Fram Strait (> 4000 m) (Figs. 2 and 3, and 4). This hotspot was used by almost all individuals instrumented in our study over eleven months of the year (not in September). It is possible this was due to the timing and location of instrumentation (typically in September on the EGS) together with deployment durations. However, this month also corresponds to the sea ice extent minimum, which may have affected use of this area. This hotspot was largely associated with residency behaviour, although some level of travel did occur within it. Although deep-water habitats are sometimes occupied by whales from other populations, they are typically not as deep as the Fram Strait (e.g., Cumberland Sound^87^ and tend to not correspond to core foraging habitats (encountered during migration or travel)^62,66,74^. It has previously been hypothesized that the bowhead whale may be a coastal, shallow water species that is only forced offshore in the presence of fast ice or due to recent ocean warming^62^. This is evidently not the case for EGSB bowhead whales, highlighting significant differences in preferred habitat characteristics among populations.

The hydrographic conditions that characterize the deep-water habitat of EGSB bowhead whales are also unique. The convergence of Atlantic Water and Arctic Water drives recirculation patterns in this region and results in localised barotropic instability^88^ and eddy formation^89^ (Fig. S1). This recirculation promotes secondary productivity of Arctic zooplankton species (*C. hyperboreus* and *C. glacialis*) in this and adjacent areas through mixing of nutrient-rich Atlantic Water with Arctic Water. Incoming Atlantic Water also contains advected boreal zooplankton species (*C. finmarchicus*)^30^, which are documented prey for other bowhead whale populations^31^, and produce a level of biomass that far exceeds local production^90^. These recirculation patterns not only promote productivity, but also physically aggregate prey and should therefore improve the foraging efficiency of bowhead whales^91^. As Atlantic Water is subducted beneath Arctic Water (the East Greenland Polar Front), vertical mixing enhances primary production and concentrates phytoplankton along frontal boundaries^92^. This subduction process maintains relatively cool SSTs (associated with habitat selection) and produces greater negative deviations in sea surface height (associated with apparent foraging). Recirculation of Atlantic Water is largely confined to south of 80 ºN^93^, consistent with the distribution of apparent foraging by instrumented whales in our study (Fig. 2). Oceanic fronts serve as important foraging habitat for many marine predators^94^, including related ram filter feeders such as southern (*Eubalaena australis*) and North Atlantic right whales (*Eubalaena glacialis*)^95,96^.

The offshore hotspot was also important habitat for EGSB bowhead whales during the reproductive season. Based on the temporal overlap in use of the two hotspots in and adjacent to the Fram Strait during this period, we suspect that foraging and reproductive activity occur in both areas, however further, finer-scale behavioural analyses are needed to confirm this hypothesis.

The dynamic environmental conditions that make this core foraging area profitable also render it, and this population of bowhead whales, vulnerable to the effects of Atlantification. As Atlantification increases the volume and temperature of Atlantic Water inflow^97^ and decreases the Arctic Water freshwater transport^27^, thermal gradients and the strength of circulation patterns between these water masses are expected to weaken^24^. These changes will almost certainly shift the location of this oceanic front, which has already been documented in recent years^27^. In the Southern Ocean, climatic anomalies have shifted the polar frontal zone, an important foraging habitat for marine predators such as king penguins (*Aptenodytes patagonicus*). Although penguins were able to track the location of the front, the vertical restructuring of prey availability increased dive effort and ultimately reduced breeding success^98^, highlighting the complexity of predicting how such changes will affect predator foraging behaviour and population demographics. Although this core area is currently largely protected by dense sea ice cover, the Arctic is projected to be seasonally ice-free in coming decades^20^. This area is particularly susceptible as the presence of eddies enhances local sea ice melting. These environmental changes will expose the EGSB bowhead whales’ core habitat, and an important reproductive habitat, to the open ocean.

### Mechanisms of resilience to climate change

Instrumented EGSB bowhead whales in our study exhibited considerable individual variability in movement patterns and foraging strategies. This variability included differences in habitat use, where some travelled between FJL while others remained on the EGS, timing and direction of long-distance travel between these regions, inconsistency in movement patterns over the EGS (locations tended to be at slightly higher latitudes in winter (Fig. S10), but directional patterns were not pervasive), differences in the way that environmental features were used (such as opportunistic foraging when travelling along the continental shelf break), and most (but not all) individuals using deep offshore habitat.

The observed intrapopulation variability suggests that this population is not migratory in the classical sense and that resource partitioning may be occurring. It is possible this resource partitioning may serve to reduce intraspecific competition, which is advantageous when resources are patchily-distributed and/or ephemeral. In the Antarctic, an ecosystem that experiences similar spatio-temporal fluxes in resource availability, sympatric krill-eating mysticete species commonly demonstrate resource partitioning^99^. These patterns may be a relic of increased competition at high population sizes prior to commercial whaling^14,100^.

As climate change continues to alter the availability and accessibility of resources across this ecosystem and decreases preferred foraging habitat for bowhead whales, this individual variability may provide resilience at the population scale^9^. In penguin populations occupying the Antarctic Peninsula, plasticity in foraging behaviour has resulted in increasing gentoo penguin populations (climate winners), while the more specialised Adélie and chinstrap penguins have had declining populations (climate losers)^101^. Although bowhead whales from the BCB and ECWG populations generally perform directed seasonal migrations (i.e., north-south or east-west)^62,84,102^, the actual routes travelled are somewhat variable for ECWG bowheads due to the complex topography and variability in oceanographic features across their range^62^, much like the movement patterns observed in our study. While our study includes the largest sample size of instrumented individuals from this population to date, it is still a small sample size in a population context (approximately 10% of the Barents Sea survey estimate^15^. Nevertheless, the considerable individual variability in habitat use and movement patterns, and presumably foraging behaviour, observed here might buffer the negative effects of climate change and enhance resilience at the population scale.

While the home range of EGSB bowhead whales did not extend north into the deep Arctic Ocean, one individual (GW21-01) travelled to 85.44 ºN (departing from the EGS, east of the Gakkel Ridge) (Fig. 2). However, it immediately turned around once it met the slope of the Amundsen Basin (> 4000 m) and returned south. Locations were predominantly inferred as travel, suggesting that suitable foraging habitat was likely not encountered. As bowhead whale habitat use and apparent foraging was inferred to occur in continental shelf/slope regions and at the interface between Atlantic and Arctic Waters, northward range expansion into deep Arctic basins seems improbable. This is supported by persistent travel by the one individual who used this habitat. Nevertheless, the use of the deep-water core foraging habitat is encouraging and suggests the suitability of deep-water habitat is primarily shaped by dynamic conditions and the accessibility of preferred prey. Changes in the distribution of zooplanktonic prey have already occurred as a result of climate change (e.g., *C. glacialis* has shifted its range poleward^103^, northward advection of *C. finmarchicus*^29)^, such that this area could potentially become more suitable habitat in the future.

Recent studies indicate that the migratory patterns of BCB and ECWG bowhead whales are changing in response to diminishing sea ice and altered oceanographic conditions. Reductions in sea ice cover have allowed bowhead whales from these populations to spend extended periods in their summer foraging grounds, shifted winter distributions northward, and altered the phenology of seasonal migrations^42,52,57,104,105^. Some individuals, both from the BCB^106^ and ECWG^87^ populations, have become non-migratory, remaining in summer foraging grounds year-around. Seasonal range shifts and behavioural plasticity^107^, more similar to the observed movement patterns of EGSB bowhead whales, appear to provide sufficient resilience to present levels of environmental change, evidenced by increased population sizes and improved body condition in these other populations^75^.

The EGSB population of bowhead whales offers a unique opportunity to study the intrinsic and extrinsic vulnerability of a small marine mammal population in an ecosystem that differs drastically from historical baselines and is also experiencing rapid environmental change. This work also provides context for how geographically separated populations, particularly in specialist species, may have different sources of vulnerability and thus respond differently to shared pressures depending on their specific habitat characteristics, and perhaps also relative population sizes. Understanding the resilience of the EGSB bowhead whale population (and other species facing similar challenges) is critical for assessing management needs that might promote recovery in ecosystems rapidly being altered by climate change.

## Supplementary Information

Below is the link to the electronic supplementary material.

Supplementary Material 1

Supplementary Material 2

## Data Availability

Data will be made available upon reasonable request (contact KM Kovacs kit.kovacs@npolar.no).
