# Supplementary material for: Endangered bowhead whales might buffer climate change with individual variability in movement patterns
Source: Sci Rep. 2026 Jan 27;16:6309. doi: 10.1038/s41598-026-36908-1 (PMC12905287; doi:10.1038/s41598-026-36908-1)
Supplement: Supplementary file 2 — Supplementary Material 2 [file 41598_2026_36908_MOESM2_ESM.docx]

**SUPPLEMENTARY information**

**Endangered bowhead whales might buffer climate change with individual variability in movement patterns**

**SUPPLEMENTARY TEXT**

**Bowhead whale data collection**

We used a helicopter (Écureuil Euroco-per AS350) platform based on a research vessel (RV Lance or RV Kronprins Haakon) that travelled deep into drift ice areas to search for, approach, and deploy instruments on bowhead whales. Instruments were deployed using an ARTS (Aerial Rocket Transmitter System) air gun (12-14 bar pressure) from a distance of approximately 10 m. The SPOT tags included one 1.5 AA-lithium battery together with the electronics in a stainless-steel casing (tube: 110 mm × 22 mm) with a stainless-steel stop-plate (38 mm in diameter) 3 cm from the distal end. The stop plate prevents the tag from penetrating too deeply into the blubber. The stainless-steel tube was attached to an anchor spear equipped with a sharp pointed tip and foldable barbs along the spear. The total length of the tag from the stop plate to the tip of the anchor was 170 mm and the weight of the instrument, with attachment spear, was 133 g. The SPLASH tags also included one 1.5 AA-lithium battery together with electronics in a stainless-steel casing and an anchoring spear equipped with a sharp tip and foldable barbs along the length of the spear. This casing was 24 mm in diameter and the whole tag from the stop plate to the tip was 300 mm and the weight of the instrument was 390 g. The LIMPET tag (56 mm x 50 mm x 27 mm) weighed 69 g and was attached to the whale with two 68 mm long 6-petals titanium dart anchors weighing 6 g each. SLDTs were surgically sterilized and the anchoring system was coated with Gentamicyn sulfate antibiotic prior to implantation.

All data were remotely transmitted via the Argos satellite system ([www.argos-system.org](http://www.argos-system.org)). SPOT and SPLASH tags were programmed to transmit Argos location data. SPOT tags were programmed to begin Argos transmission attempts at 06:00 h each day while SPLASH tags began transmission attempts at 00:00 h. All tags were programmed to transmit up to 200 times per day and to transmit data collected over the last 7 days. The LIMPET tag was programmed to transmit both Argos (using the same programming as SPLASH tags) and Fastloc GPS data, attempting Fastloc GPS transmissions after every 60 min when the animal surfaced. A maximum of four successful Fastloc GPS transmission attempts per hour (30 per day) and four failed attempts per hour were allowed, for a maximum of 100 overall attempts per day.

Skin biopsies were collected after instrument deployment when feasible, using a crossbow and custom-made arrows with a biopsy dart at the tip (4 cm in length and 8 mm in diameter) which bounced off the whale and floated at the surface. Samples were retrieved using a small hoop net attached to a telescopic pole while the helicopter hovered close to the sea surface. Skin samples were frozen at -20 ºC upon return to the ship until analysis.

**Location data filtering**

A coarse distance filter (great circle distance of 100 km from the previous location or to the following location) was used to remove implausible locations, account for inaccurate starting locations, and filter clusters of locations with more than one location from the expected path^1,2^. Individual tracks were separated into track segments if there was a gap in transmission greater than 48 h prior to model fitting. Track segments were then filtered to remove duplicate dates with a minimum of 10 s between subsequent locations, locations with swim speeds greater than 2 m s^-1^, and locations requiring turning angles less than 15 degrees if the distance leading to the location was greater than 2,500 m or requiring turning angles less than 25 degrees if the distance leading to the location was greater than 5,000 m. Two additional locations were manually removed from the beginning of one track segment that resulted in unrealistic predicted locations (high horizontal displacement speeds).

**Environmental data collection**

All environmental data were assigned to location data by the nearest available date, based on geographic coordinates, unless otherwise specified.

1. **Bathymetric data**

Bathymetric data were obtained from the NOAA ETOPO 2022 database (<https://doi.org/10.25921/fd45-gt74>) at a resolution of 4 arc minutes (1/60 degree) using the R package *marmap* version 1.0.10 and matched to location data^3^. Given the resolution of the data and close proximity of some locations to shore, locations with depth values less than -4 m (i.e., shallower than 4 m below sea level) were assigned values of -4 m. This depth was chosen based on the body size of bowhead whales. Bathymetric data were then used to calculate the great circle distance (km) locations to the nearest 500 m isobath which reflects the approximate distance to the continental shelf break. We then rasterized bathymetric data to calculate the slope of the sea floor within each cell in degrees using eight neighbouring cells (suitable for rough surfaces) to compute the topographical slope for each cell.

1. **Sea Ice**

Daily sea ice extent data were downloaded from the NSIDC Sea Ice Index, Version 3 (<https://doi.org/10.7265/N5K072F8>) at a 25 km x 25 km spatial resolution and were used to determine whether locations were inside the ice edge, where the ice extent included any cell with a concentration of 15% or greater. Given the prevalence of sea ice outside the main ice edge and open water areas inside the main ice edge, sea ice data and water data were cast to polygons. The sea ice polygon with the largest area of sea ice was considered the main ice edge and any holes in this polygon (i.e., polynyas) were then filled in using the R package *nngeo* version 0.4.8^4^. Locations were then assigned to one of two categories – either inside or outside the main ice edge.

Daily sea ice concentration data at a spatial resolution of 25 km x 25 km were acquired from the Nimbus-7 SMMR and DMSP SSM/I-SSMIS Passive Microwave Data, Version 2 (<https://doi.org/10.5067/MPYG15WAA4WX>) and values were assigned corresponding to the nearest date and geographic coordinates for each location and simulated location. Rasterized daily sea ice concentration data were used to determine contour lines for ice edge using the threshold of 15% (corresponding to the marginal ice zone) for each day. Using a raster of bathymetric data at a resolution of 10 min (low resolution) to improve computational efficiency, we calculated a transition matrix for land and ocean, whereby land was impassible to bowhead whales. We then used this transition matrix with the R package *gdistance* version 1.6.4 to calculate the least cost distance of each bowhead whale location to the nearest 15% contour line for that day (including polynyas inside the main ice edge) rerouting each path around land^5^. A small number of near-shore locations were classified as on shore (n = 16) and so were assigned distance to ice edge values of the location preceding it temporally. Visual inspection was used to confirm that these locations were approximately overlapping.

To determine whether bowhead locations were located within polynyas, we first determined whether locations were inside the main ice edge using the methods described above. Daily sea ice extent data were downloaded from the NSIDC Sea Ice Index, Version 3 (<https://doi.org/10.7265/N5K072F8>) at a 25 km x 25 km spatial resolution and used to determine whether locations were in open water. Open water included any cell with less than 15% ice concentration which were cast to polygons to represent open water areas. Two criteria were used to identify whether locations were in polynyas (i) if they were both inside the main ice edge and located in open water or (ii) if they were both inside the main ice edge and in a cell with sea ice concentration less than 15%. Based on these criteria, few locations (n = 110) were considered to be located inside polynyas.

1. **Dynamic oceanographic conditions**

Monthly mean values of sea water potential temperature at 0 m depth (hereby referred to as sea surface temperature or SST, ºC), barotropic streamfunction (m^3^ s^-1^), and sea surface height above geoid (m) were acquired from the Copernicus Arctic Ocean Physics Reanalysis (<https://doi.org/10.48670/moi-00007>) at a 12.5 x 12.5 km resolution. These data were assigned to each location based on the corresponding month and their geographic coordinates.

Daily eastward ocean current velocity (*uo*, m s^-1^) and northward ocean current velocity (*vo*, m s^-1^) data were taken from the Copernicus Global Ocean Reanalysis ((<https://doi.org/10.48670/moi-00021>) at 0.5 m (depth nearest surface) at a spatial resolution of 0.083º x 0.083º resolution. Data were rasterized and the mean *uo* and *vo* for each grid cell were calculated and used to calculate eddy kinetic energy (EKE, m^2^ s^-2^) for each grid cell using the following equation:

EKE = ½ (*u*’^2^+ *v’^2^*) (Equation S1)

where *u*’ represents the daily eastward ocean current velocity anomaly (*u* – *u_mean_*) and *v*’ represents the daily northward ocean current velocity anomaly (*v* - *v_mean_*) for that grid cell. We calculated the seasonal mean EKE using daily EKE values for each grid cell between September 2021 and August 2022 to explore the spatio-temporal variability in EKE across core bowhead whale habitat over the period in which most location data were available (Table 1). We took a conservative approach and did not include eddy kinetic energy in analyses because it was estimated using other oceanographic variables that were generated from oceanographic models.

1. **Marine-terminating glacier fronts**

Data were available for Greenland (<https://doi.org/10.7280/D1667W>) and Svalbard (<https://doi.org/10.21334/NPOLAR.2021.D60A919A>) to calculate the proximity of bowhead whale locations to marine-terminating glacier fronts. Data for Greenland were filtered to include data between 2017 and 2022 (our study years) and data for Svalbard was taken from 2017 (the first year of our study). Polygon data for Greenland were simplified to retain 0.001% of points and 0.01% for Svalbard to improve computational efficiency and reduce redundancy in calculations. Outlines of all glaciers for Franz Josef Land (Russia) were acquired from the Randolph Glacier Inventory (<https://doi.org/10.7265/4m1f-gd79>) which were filtered to retain 0.01% of points. The depth at each location (see bathymetric data) was used to determine if each glacier was marine-terminating (i.e., located over the ocean). We then used the transition matrix used in calculating distance to sea ice edge to determine the shortest straight line (least cost) distance for each bowhead whale location to the nearest marine-terminating glacier front rerouting paths around land.

**S 2 Environmental data transformations**

Environmental predictors included in the modified resource selection function and linear mixed-effects modelling were transformed prior to model fitting for each model type separately to improve model fit and convergence. To facilitate interpretation of the influence of topographical features and accommodate non-linear effects given the bimodal nature of depth data (peaks corresponding to the continental shelf and deep-water habitat), the absolute value of depth and a natural cubic spline with knots at 220 m, 500 m, and 2500 m were used. Knots were based on quantiles of the depth data including simulated data (218.8 m, 495.1 m, 2737.5 m) and depths associated with distinct topographical features across the bowhead whale habitat (i.e., continental shelf banks, the continental shelf break, and deep-water basins, respectively). The absolute minimum value of sea surface temperature (-1.902 including simulated data, -1.898 including only re-routed location data) + 0.001 was added to all sea surface temperatures which were then log-transformed. Sea ice concentration was re-scaled between 0.025 and 0.975 to exclude 0 and 1 and then logit-transformed. The square of transformed sea ice concentration was also included to allow for relationships with intermediate sea ice concentrations. As continuous variables, the distance to the shelf break, distance to the ice edge, and distance to marine terminating glacier fronts were all log-transformed; to permit log-transformation, 0.001 was added to distance to the ice edge and distance to glacier fronts as some locations overlapped with these features. To facilitate interpretation the sea surface height above geoid and because all values were less than 0, the absolute value was log-transformed.

**Statistical challenges and limitations**

We encountered some challenges associated with fitting either random walk or correlated random walk models to all track segments. This is likely related to the behaviour of the whales, whereby they are in ice-covered areas much of their time and often breathe in narrow leads (i.e., instrument not surfacing during respiration in dense sea ice). Although location data were collected relatively frequently (median time between locations of 0.7 h), longer gaps were still common in the data (mean and standard deviation for time between locations of 1.7 ± 3.7 h). For Argos data, 92.03% of locations were considered low-quality (i.e., class B, A, or 0), some of which were highly implausible and thus removed during data filtering. Random walk models tend not to deal well with small to moderate gaps (relative to the predictive time step) and over-fit to particularly noisy tracks^6^. However, filtering our data to reduce noise and separating tracks into track segments mitigated these issues for most tracks as locations were generally close together temporally.

While the correlated random walk model typically produces a better fit over small to moderate gaps, it can also estimate unlikely movement patterns through longer gaps and result in over-smoothing^6^, which was the case for our data. The correlated random walk model only fit better for tracks which had short durations and a relatively high number of gaps, as expected. Another possible explanation for why the random walk model fit best to most tracks is the affiliation of bowhead whales for sea ice. The movement patterns of bowhead whales tend to follow open water areas in the sea ice (i.e., ponds or leads) to reduce the energetic costs and risks (i.e., entrapment) associated with breaking through dense sea ice to breathe. Thus, preferred habitat is not uniformly distributed in space or correlated in direction, even on relatively small spatial scales.

A predictive time-step of four hours was selected based on several criteria including the time interval between locations following filtering and separation into track segments (i.e., mean of at least one location between each predictive time step), assessment of state-space model fits, and comparison of move persistence estimates along track segments. Although state-space models fitted well at shorter time steps (e.g., two or three hours), move persistence estimates fitted to track segments predicted at shorter time steps were strongly biased toward travel and had high standard error estimates. This was expected given that behaviour of bowhead whales inferred as foraging elsewhere may last days to months^7^. As ram filter feeders, bowhead whales generally move slowly (median horizontal displacement speed of 0.26 m s^-1^) and with low tortuosity, resulting in over-estimation of high move persistence at low time steps. The low horizontal displacement speed between successive movement locations and directional persistence occurring below the patch scale could not be sufficiently resolved at shorter time steps without the inclusion of additional information, such as dive data. Simulations from state-space models with starting locations near small islands near Franz Josef Land resulted in simulated track segments for one animal suggesting that it consistently moved northward due to the gradient raster including land as a barrier to whale movement, which may have biased simulated locations for this individual (Fig. S5).

A limitation of this modelling framework is the inability to account for multiple behaviours that may be characterized by similar movement parameters (e.g., apparent foraging, rest, or reproductive behaviour) when using move persistence estimates to infer behaviour. Given the chosen time step, consistently low horizontal displacement speed, and number of consecutive locations with low or high move persistence estimates, we expect that bowhead whales sleep during behaviours inferred as both foraging and travel^8^. A similar concern is that the prevalence of low move persistence during the reproductive season may bias the importance of this habitat. However, recently published research suggests bowhead whales forage year-round^9^ and therefore we expect the environmental and prey conditions in breeding areas make them suitable foraging habitat as well. Future work should consider the influence of other behavioural modes on the spatio-temporal distribution of this population and the inclusion of other data streams if possible.

**SUPPLEMENTARY TABLES**

**Table S1** Individual EGSB bowhead whale satellite-linked data transmitter (SLDT) deployment records. Data include sex, deployment date, first and last transmission dates, tracking duration between first and last transmission dates, and the total number of tracking days (the number of days with at least one location transmission).

|  | Year | Individual | SLDT Model | Sex | Deployment Date | | First Transmission | Last Transmission | Tracking Duration | Tracking Days |
| --- | --- | --- | --- | --- | --- | --- | --- | --- | --- | --- |
| 1 | 2017 | GW17-01 | SPOT | - | 2017-05-30 | | 2017-05-30 14:49:05 | 2017-06-08 06:06:55 | 8.64 | 10 |
| 2 | 2017 | GW17-02 | SPOT | - | 2017-06-01 | | 2017-06-03 14:40:27 | 2018-03-14 17:08:53 | 284.10 | 173 |
| 3 | 2017 | GW17-03 | SPOT | - | 2017-06-01 | | 2017-06-03 11:18:02 | 2017-06-06 08:36:01 | 2.89 | 4 |
| 4 | 2017 | GW17-04 | SPOT | - | 2017-06-02 | | 2017-06-02 12:26:51 | 2017-06-06 17:22:45 | 4.21 | 5 |
| 5 | 2017 | GW17-05 | SPOT | M | 2017-06-03 | | 2017-06-07 06:22:10 | 2018-03-11 06:46:30 | 277.02 | 230 |
| 6 | 2017 | GW17-06 | SPOT | - | 2017-06-04 | | 2017-06-04 07:45:17 | 2017-11-08 16:53:35 | 157.38 | 157 |
| 7 | 2017 | GW17-07 | SPOT | F | 2017-06-04 | | 2017-06-06 15:46:01 | 2017-10-05 14:21:52 | 120.94 | 57 |
| 8 | 2017 | GW17-08 | SPOT | M | 2017-06-04 | | 2017-06-04 19:24:15 | 2018-01-14 14:43:04 | 223.80 | 166 |
| 9 | 2017 | GW17-09 | SPOT | - | 2017-06-04 | | 2017-06-23 06:25:49 | 2017-09-12 22:46:14 | 81.68 | 82 |
| 10 | 2017 | GW17-10 | SPOT | F | 2017-06-04 | | 2018-05-23 06:08:39 | 2018-05-31 07:15:29 | 8.05 | 9 |
| 11 | 2017 | GW17-11 | SPOT | - | 2017-06-04 | | 2017-07-23 10:06:11 | 2017-08-30 17:59:03 | 38.33 | 36 |
| 12 | 2017 | GW17-13 | SPOT | - | 2017-06-04 | | 2017-06-05 07:35:48 | 2017-06-10 22:14:51 | 5.61 | 6 |
| 13 | 2017 | GW17-15 | SPOT | - | 2017-06-05 | | 2017-08-29 14:32:52 | 2019-05-23 18:00:43 | 632.14 | 263 |
| 14 | 2018 | GW18-01 | SPOT | - | 2018-08-31 | | 2018-09-19 07:31:09 | 2019-11-18 14:45:29 | 425.30 | 217 |
| 15 | 2018 | GW18-02 | SPOT | - | 2018-08-31 | | 2018-10-18 08:42:49 | 2018-10-29 13:57:54 | 11.22 | 11 |
| 16 | 2018 | GW18-03 | SPOT | - | 2018-08-31 | | 2018-08-31 11:09:44 | 2018-10-17 17:13:13 | 47.25 | 21 |
| 17 | 2018 | GW18-04 | SPOT | - | 2018-08-31 | | 2018-09-03 08:02:51 | 2018-12-29 08:15:26 | 117.01 | 115 |
| 18 | 2018 | GW18-07 | SPOT | - | 2018-09-03 | | 2019-01-14 20:04:43 | 2019-09-27 08:38:16 | 255.52 | 175 |
| 19 | 2018 | GW18-08 | SPOT | - | 2018-09-03 | | 2018-09-04 08:47:48 | 2019-05-29 14:10:19 | 267.22 | 248 |
| 20 | 2018 | GW18-09 | SPOT | - | 2018-09-03 | | 2018-09-05 06:00:40 | 2019-03-07 12:23:33 | 183.27 | 165 |
| 21 | 2018 | GW18-10 | SPOT | - | 2018-09-03 | | 2018-11-03 11:37:10 | 2019-06-17 17:34:01 | 226.25 | 104 |
| 22 | 2018 | GW18-11 | SPOT | - | 2018-09-03 | | 2018-09-03 12:21:58 | 2019-06-20 13:42:00 | 290.06 | 221 |
| 23 | 2019 | GW19-01 | SPOT | F | 1 | 2019-09-05 | 2019-09-05 15:17:09 | 2019-10-22 11:23:19 | 46.84 | 44 |
|  |  |  | LIMPET |  | 2 | 2019-09-05 | 2019-09-05 15:40:29 | 2019-09-12 19:20:53 | 7.15 | 8 |
|  |  |  | SPOT |  | 3 | 2019-09-05 | 2019-10-08 15:12:46 | 2020-02-19 11:39:04 | 133.85 | 131 |
| 24 | 2020 | GW20-02 | SPLASH | M | 2020-09-07 | | 2020-09-07 18:36:05 | 2021-05-24 17:22:59 | 258.95 | 258 |
| 25 | 2020 | GW20-03 | SPLASH | - | 2020-09-08 | | 2021-02-05 13:50:54 | 2021-08-04 23:30:59 | 180.40 | 67 |
| 26 | 2021 | GW21-01 | SPLASH | M | 2021-08-10 | | 2021-08-10 09:33:47 | 2022-04-12 20:31:59 | 245.46 | 246 |
| 27 | 2021 | GW21-02 | SPLASH | M | 2021-08-10 | | 2021-08-11 23:10:00 | 2022-06-08 17:25:39 | 300.76 | 234 |
| 28 | 2021 | GW21-03 | SPLASH | M | 2021-08-10 | | 2021-08-10 16:13:57 | 2022-06-28 00:39:58 | 321.35 | 138 |
| 29 | 2021 | GW21-04 | SPLASH | M | 2021-08-10 | | 2021-08-14 02:48:00 | 2022-07-05 12:59:06 | 325.42 | 189 |
| 30 | 2021 | GW21-05 | SPLASH | M | 2021-08-10 | | 2021-08-10 12:12:57 | 2022-03-22 19:21:37 | 224.30 | 221 |
| 31 | 2021 | GW21-06 | SPLASH | M | 2021-08-10 | | 2021-08-12 11:07:01 | 2022-05-21 01:46:46 | 281.61 | 246 |
| 32 | 2021 | GW21-07 | SPLASH | M | 2021-08-10 | | 2021-08-19 13:33:41 | 2021-10-05 02:04:14 | 46.52 | 16 |
| 33 | 2021 | GW21-08 | SPLASH | M | 2021-08-10 | | 2021-08-10 14:27:56 | 2022-04-01 20:59:07 | 234.27 | 231 |
| 34 | 2021 | GW21-09 | SPLASH | M | 2021-08-10 | | 2021-09-29 19:03:12 | 2022-04-30 20:46:34 | 213.07 | 107 |
| 35 | 2021 | GW21-10 | SPLASH | M | 2021-08-10 | | 2022-05-30 20:50:42 | 2022-07-04 16:38:17 | 34.82 | 36 |
| 36 | 2021 | GW21-11 | SPLASH | M | 2021-08-10 | | 2021-09-12 01:05:18 | 2022-04-08 19:25:15 | 208.76 | 187 |
| 37 | 2021 | GW21-12 | SPLASH | M | 2021-08-10 | | 2021-09-10 15:05:43 | 2022-05-06 06:28:59 | 237.64 | 236 |
| 38 | 2021 | GW21-13 | SPLASH | M | 2021-08-10 | | 2021-09-29 21:51:46 | 2022-04-19 22:27:32 | 202.02 | 203 |

**Table S2** Summary of environmental covariates including their definitions, spatial and temporal resolution of data collection, whether the feature was static or dynamic, the data type, transformations used, and for which models (modified resource selection function (RSF) or linear mixed-effects model (LMM)) environmental covariates were included as predictors.

| Covariate | Definition | Spatial Resolution | Temporal Resolution | Feature Type | Data Type | Transformation | Model |
| --- | --- | --- | --- | --- | --- | --- | --- |
| dpth | Depth | 4 arc minutes  (1/60 degree) | - | Static | Continuous (m) |  | RSF, LMM |
| slp | Slope | 4 arc minutes  (1/60 degree) | - | Static | Continuous (degrees) | log | LMM |
| dst_shlf_ | Distance to shelf break  (500 m isobath) | 4 arc minutes  (1/60 degree) | - | Static | Continuous (km) | log | LMM |
| in_ice_ | Inside Ice Edge | 25 km x 25 km | Daily | - | Categorical | - | RSF, LMM |
| ice | Sea Ice Concentration | 25 km x 25 km | Daily | Dynamic | Continuous between 0 and 1 | logit | RSF, LMM |
| ice_sq_ | Transformed Sea Ice Concentration Squared | 25 km x 25 km | Daily | Dynamic | Continuous between 0 and 1 | - | RSF, LMM |
| dst | Distance to Ice Edge | 25 km x 25 km | Daily | Dynamic | Continuous (km) | log | LMM |
| polynya | Polynya | 25 km x 25 km | Daily |  | Categorical | - | - |
| sst | Sea Surface Potential Temperature at 0 m Depth | 12.5 km x 12.5 km | Monthly | Dynamic | Continuous (ºC) | log | RSF, LMM |
| baro | Barotropic Streamfunction | 12.5 km x 12.5 km | Monthly | Dynamic | Continuous (m^3^ s^-1^) | log | - |
| ssh | Sea Surface Height Above Geoid | 12.5 km x 12.5 km | Monthly | Dynamic | Continuous (m) | log | LMM |
| dst_glc_ | Distance to Glacier Front | - | - | Static | Continuous (km) | log | LMM |
| eke | Eddy Kinetic Energy at 0.5 m Depth | 0.083° × 0.083° | Daily |  | Continuous (m^2^ s^-2^) | - | - |

**Table S3** Candidate linear mixed-effects models for the relationship between environmental covariates and move persistence estimates for EGSB bowhead whale tracks. Models were fitted using Maximum Likelihood estimation. Environmental covariates include dpth as depth (m), slp (º) as sea floor slope, dst_shlf_ as distance to the shelf break (500 m isobath) (km), dst_glc_ as distance to marine-terminating glacier front, sst as sea surface temperature (ºC), ssh as sea surface height above geoid (m), dist2ice as distance to sea ice edge, sea_ice as sea ice concentration, and sea_ice_sq as sea ice concentration squared. The degrees of freedom (df), log-Likelihood (logLik), Corrected Akaike Information Criterion (AICc), ΔAICc, and weight are also presented.

**Table S4**  Number of locations predicted at a four-hour time step using state-space models separated by month that were collected by EGSB bowhead whales (n = 37) between 2017 and 2021.

| Month | Jan | Feb | Mar | Apr | May | Jun | Jul | Aug | Sep | Oct | Nov | Dec |
| --- | --- | --- | --- | --- | --- | --- | --- | --- | --- | --- | --- | --- |
| Locations | 2816 | 2149 | 2350 | 1548 | 1409 | 1720 | 1293 | 1432 | 3158 | 3933 | 2929 | 2973 |

**Table S5** Kernel utilization distribution overlap between seasons calculated using Bhattacharyya’s affinity for the (a) home range (95% utilization distribution) and (b) core areas (50% utilization distribution) for EGSB bowhead whales.

1. (b)

|  | Spring | Summer | Fall | Winter |  |  | Spring | Summer | Fall | Winter |
| --- | --- | --- | --- | --- | --- | --- | --- | --- | --- | --- |
| Spring |  |  |  |  |  | Spring |  |  |  |  |
| Summer | 0.683 |  |  |  |  | Summer | 0.267 |  |  |  |
| Fall | 0.601 | 0.496 |  |  |  | Fall | 0.258 | 0.004 |  |  |
| Winter | 0.701 | 0.468 | 0.567 |  |  | Winter | 0.418 | 0.145 | 0.301 |  |

**SUPPLEMENTARY FIGURES**

**
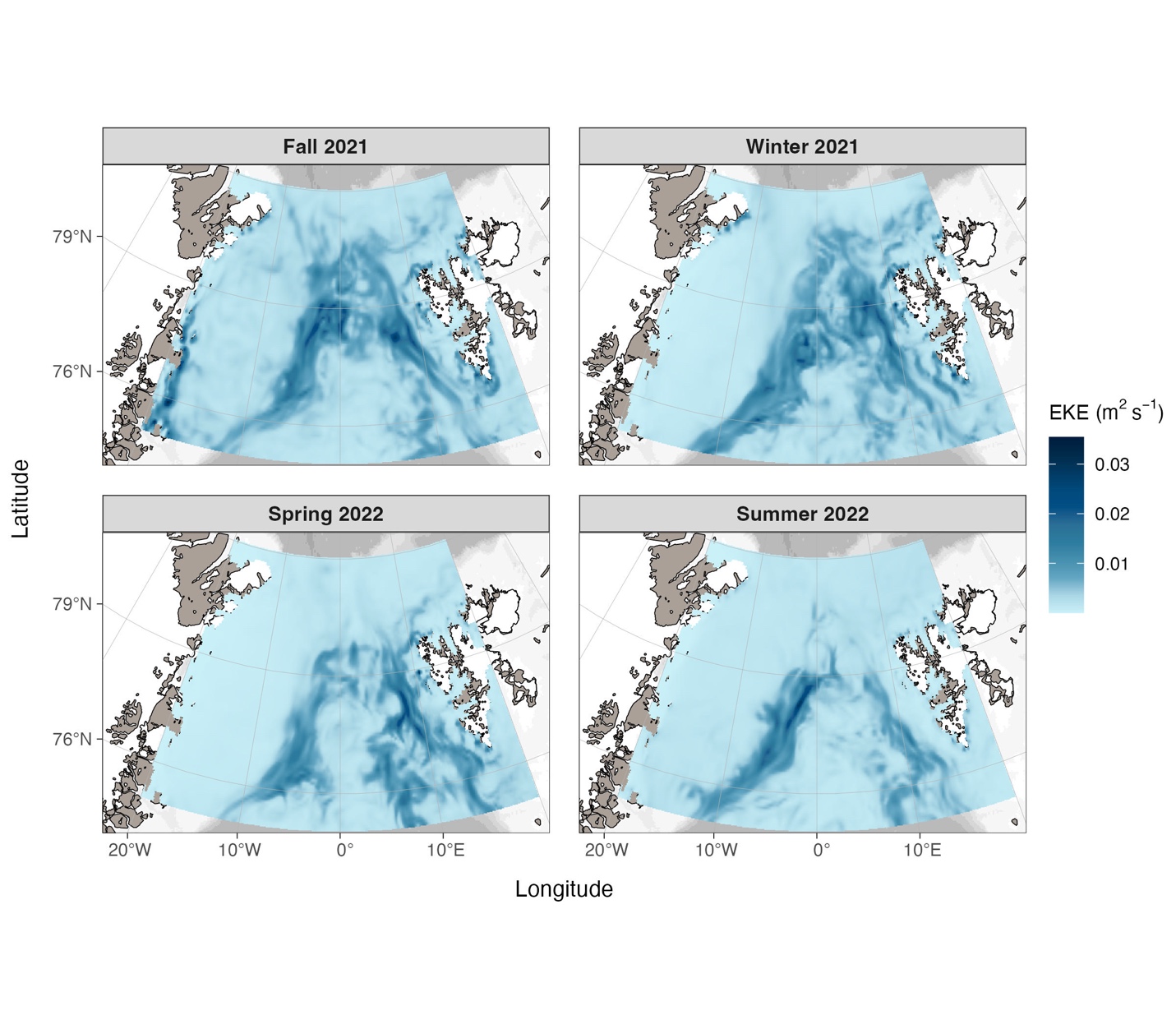
**

**Fig. S1** Map of eddy kinetic energy (EKE; m^2^ s^-2^) averaged over each season for each grid cell between fall 2021 and summer 2022 for the Fram Strait. The map was produced using the R package *ggOceanMaps*^10^.


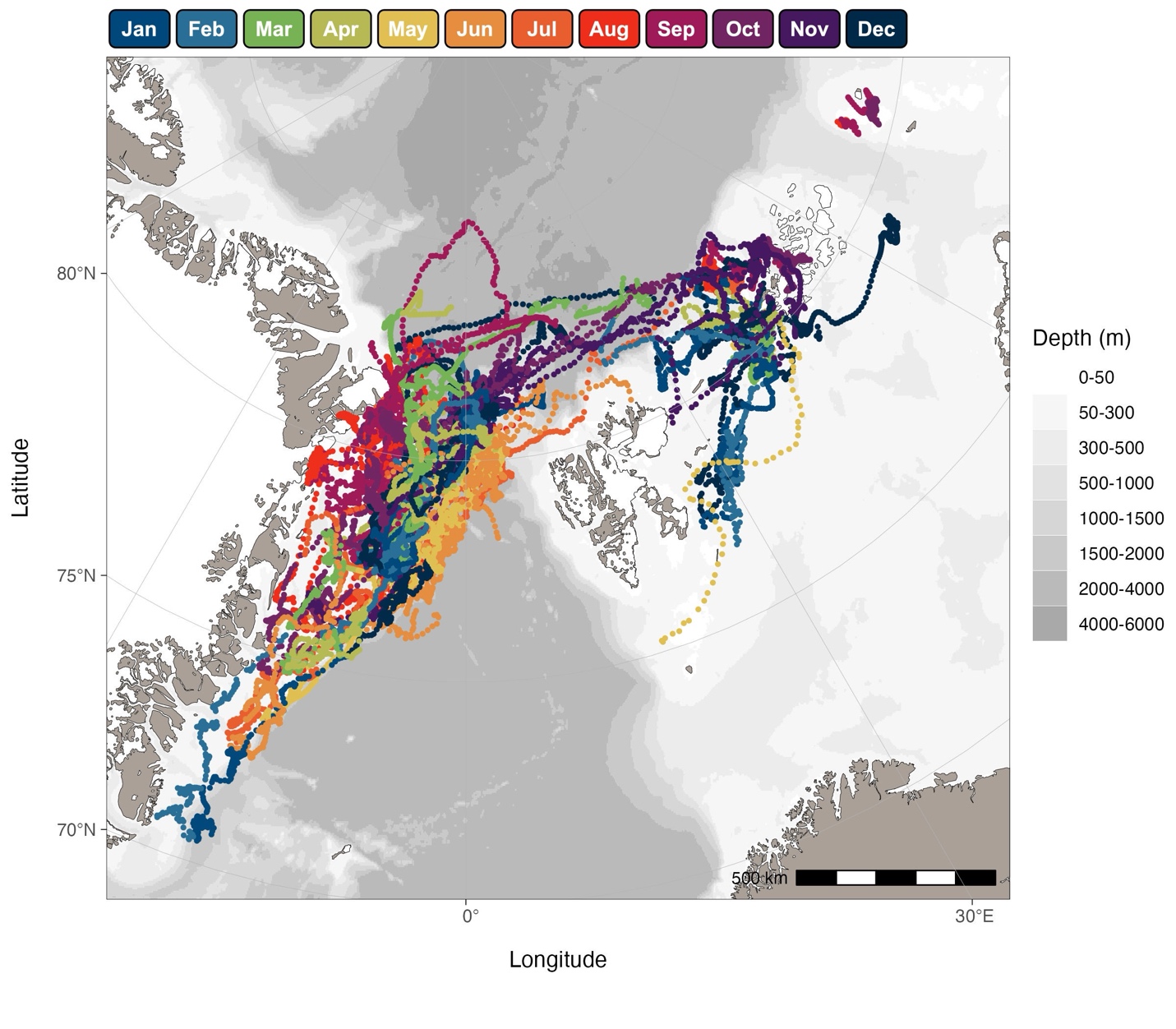


**Fig. S2** Map of all EGSB bowhead whale (n = 37) locations predicted using state-space models at a four-hour time step coloured by month. The map was produced using the R package ggOceanMaps^10^.


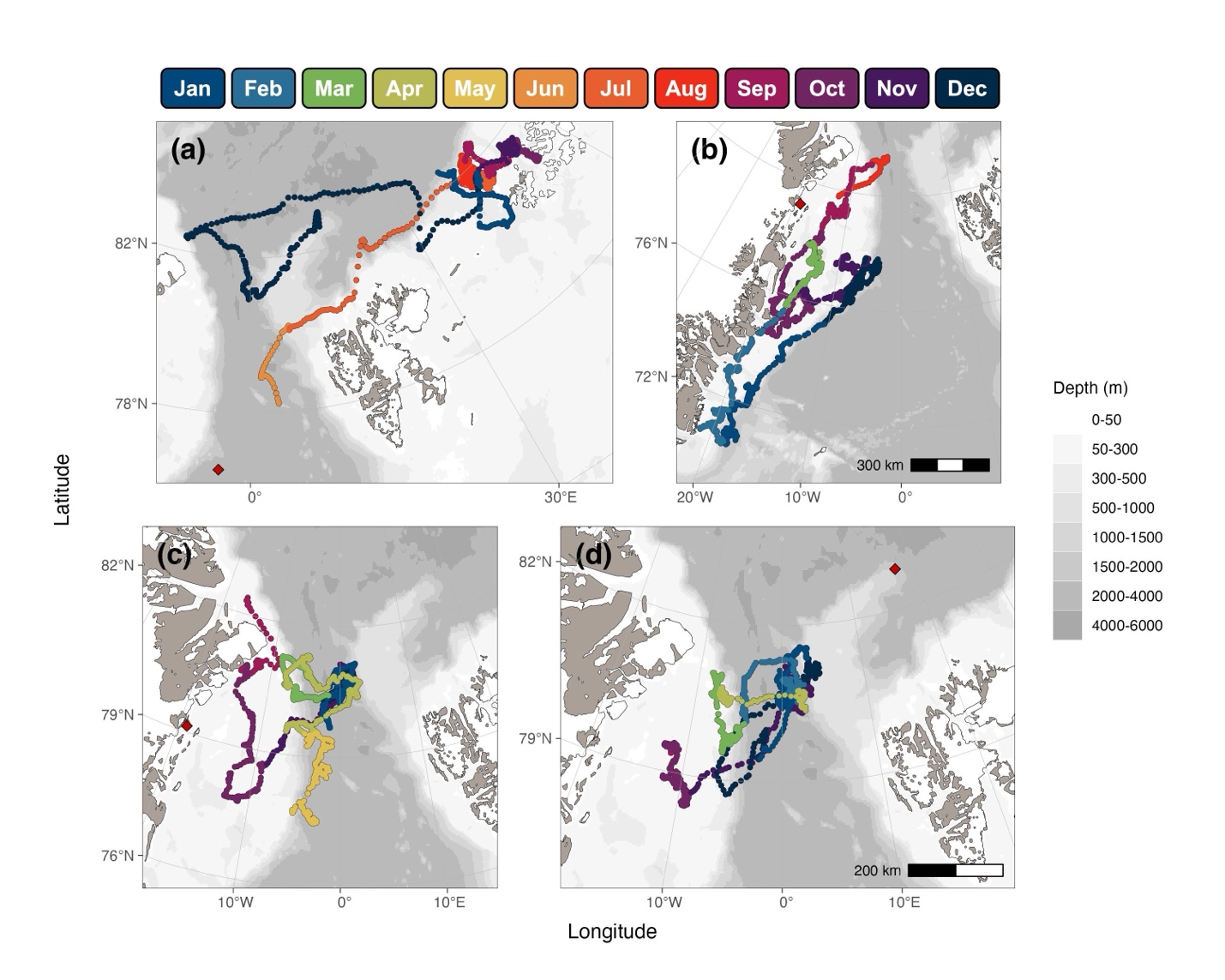


**Fig. S3** Map of EGSB bowhead whale locations coloured by month for (a) GW17-05, (b) GW21-05, (c) GW21-06, and (d) GW21-13 illustrating differences in seasonal habitat use among individuals. Deployment locations are included as red diamonds. Maps were produced using the R package *ggOceanMaps*^10^.


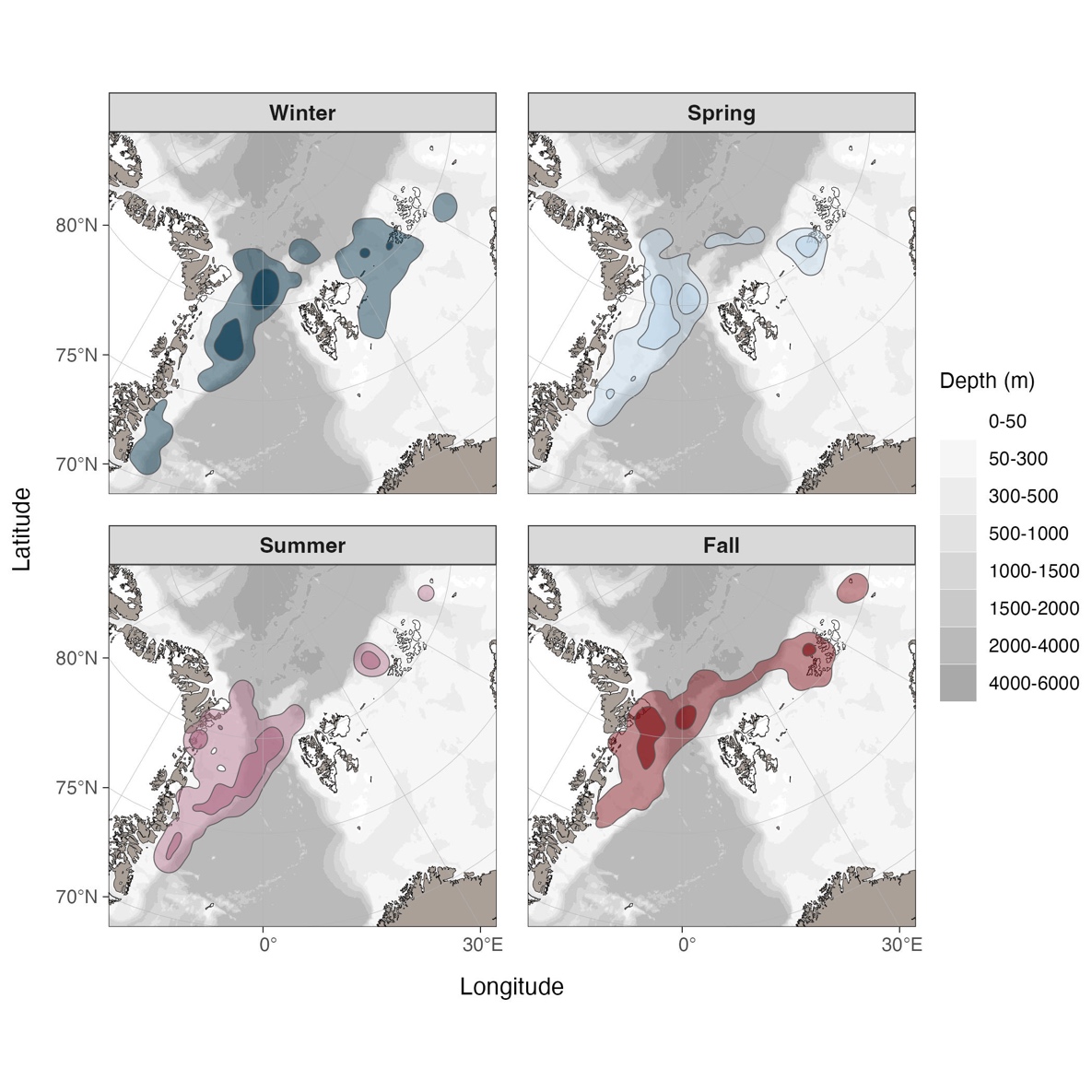


**Fig. S4** Maps of the seasonal (winter = Dec, Jan, Feb; Spring = Mar, Apr, May; Summer = Jun, Jul, Aug; Fall = Sep, Oct, Nov) home range (95% kernel utilization distribution) and core areas (50% kernel utilization distribution) of EGSB bowhead whales (n = 37). Maps were produced using the R package ggOceanMaps^10^.

**
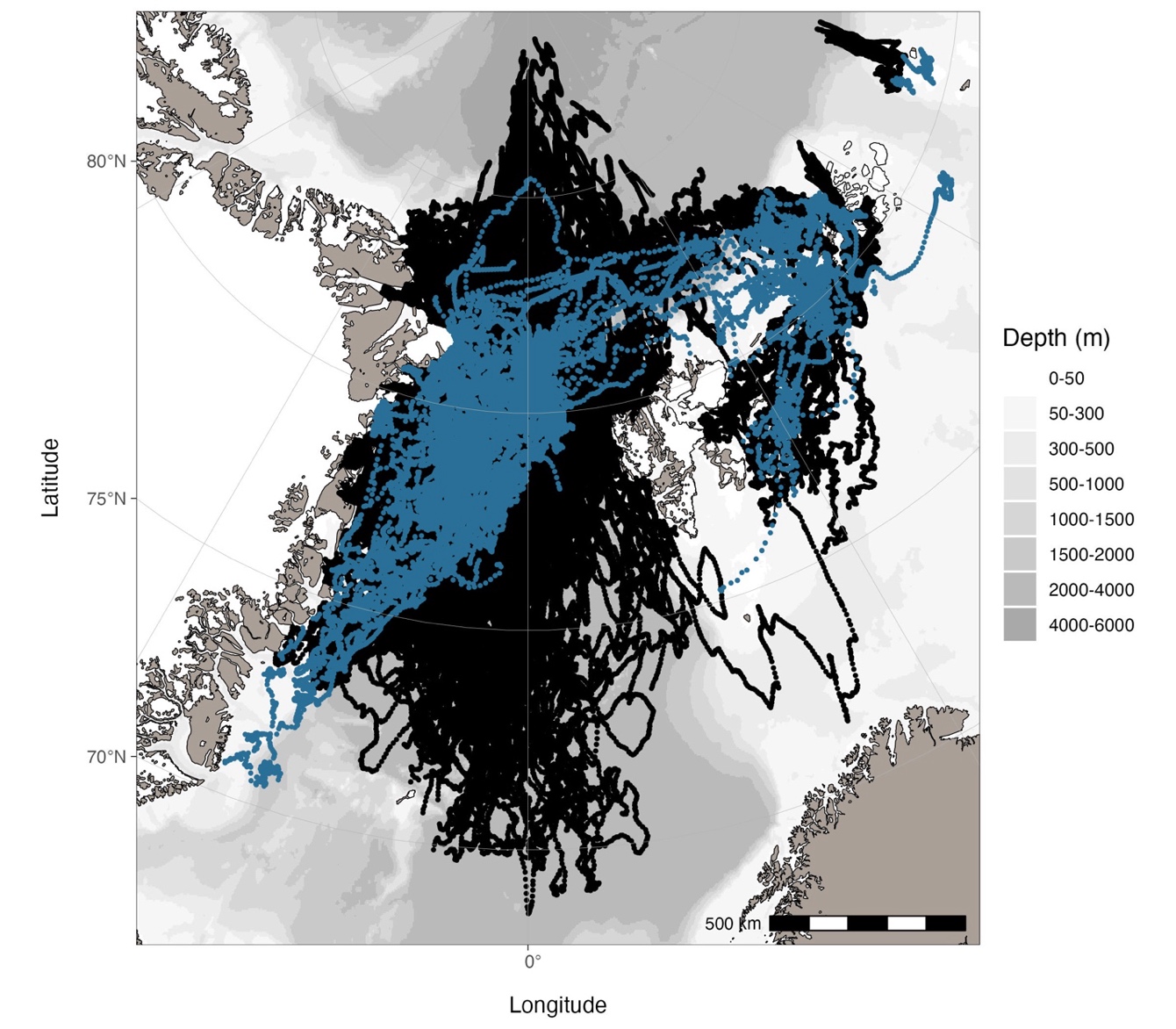
**

**Fig. S5** Map of EGSB bowhead whale (n = 37) locations predicted at a four-hour time step using state-space models considered to be the used habitat (blue) and locations simulated from the movement parameters of each state-space model (black) considered to be the available habitat. For each state-space modelled track segment, 100 track segments were simulated and a similarity filter was used to retain the best 20 which are shown here. The map was produced using the R package ggOceanMaps^10^.


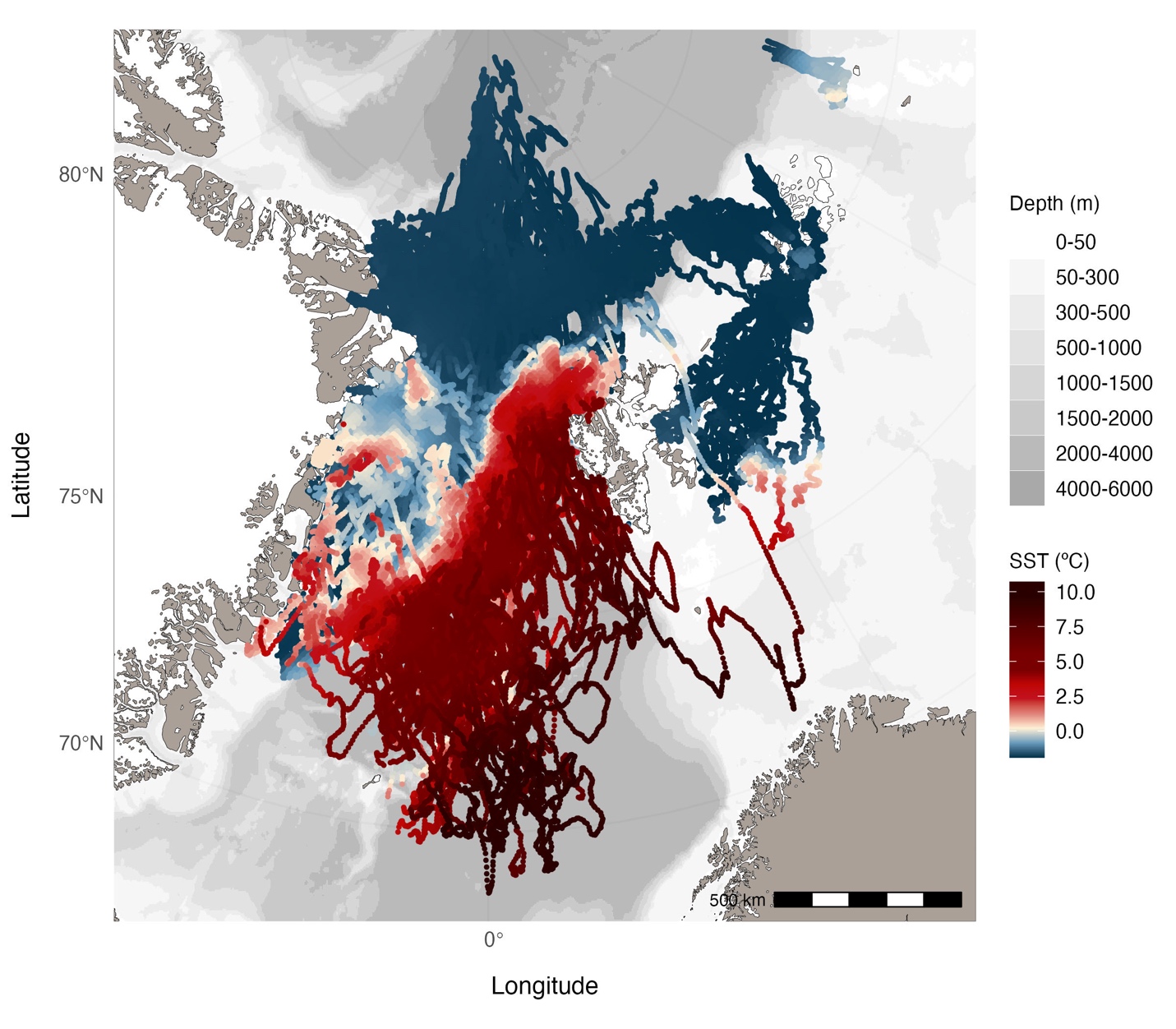


**Fig. S6** Map of sea water potential temperature at 0 m depth (also referred to as sea surface temperature; SST; ºC) at predicted and simulated EGSB bowhead whale locations. Locations were simulated from the movement parameters of state-space modelled track segments. The map was produced using the R package *ggOceanMaps*^10^.


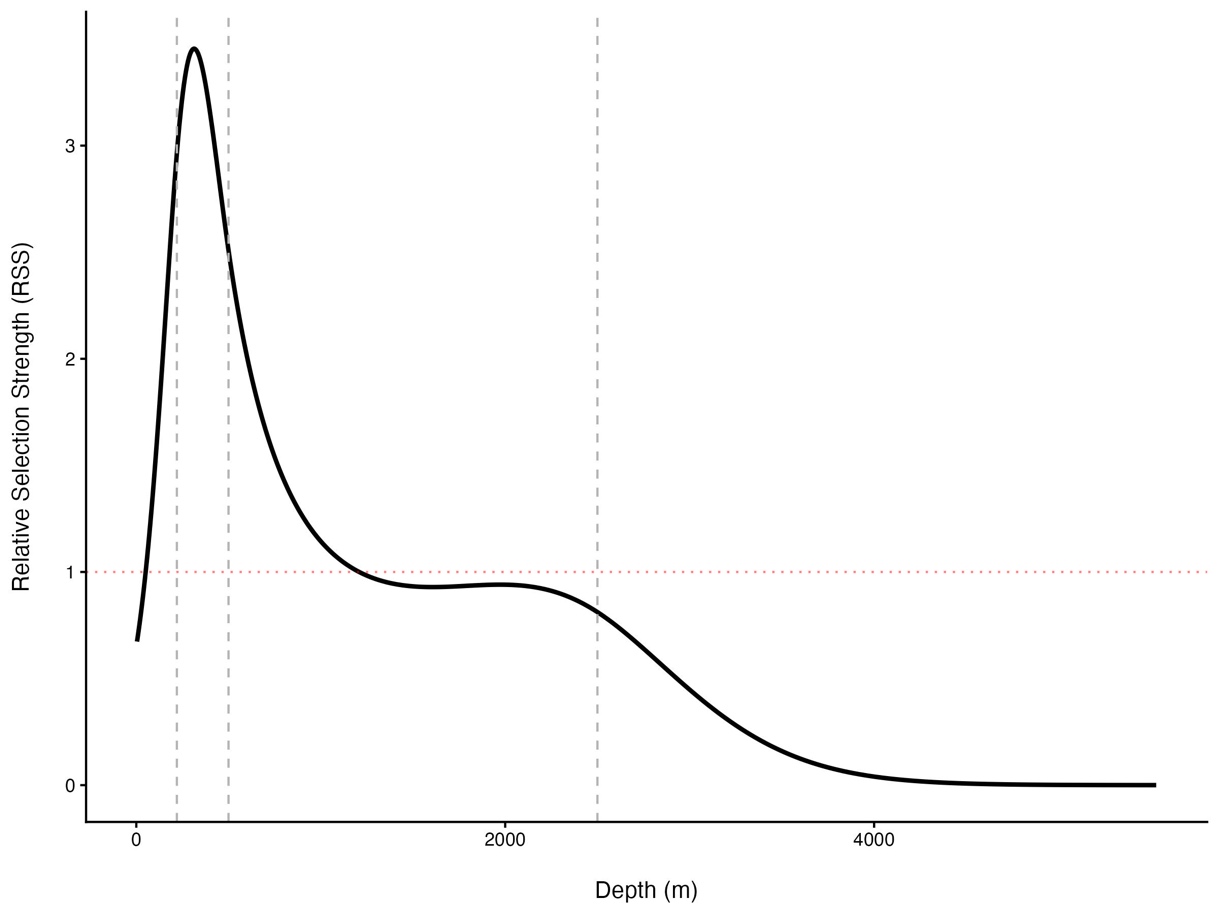


**Fig. S7** The relative selection strength (RSS) for depth (m) by bowhead whales predicted by the modified resource selection function. Values were calculated by varying depth while holding all other environmental covariates constant at their mean (standardized to zero). The red dotted line indicates neutral selection (RSS = 1). Grey dashed lines indicate topographical knots used for the natural cubic spline (220 m, 500 m, 2500 m).


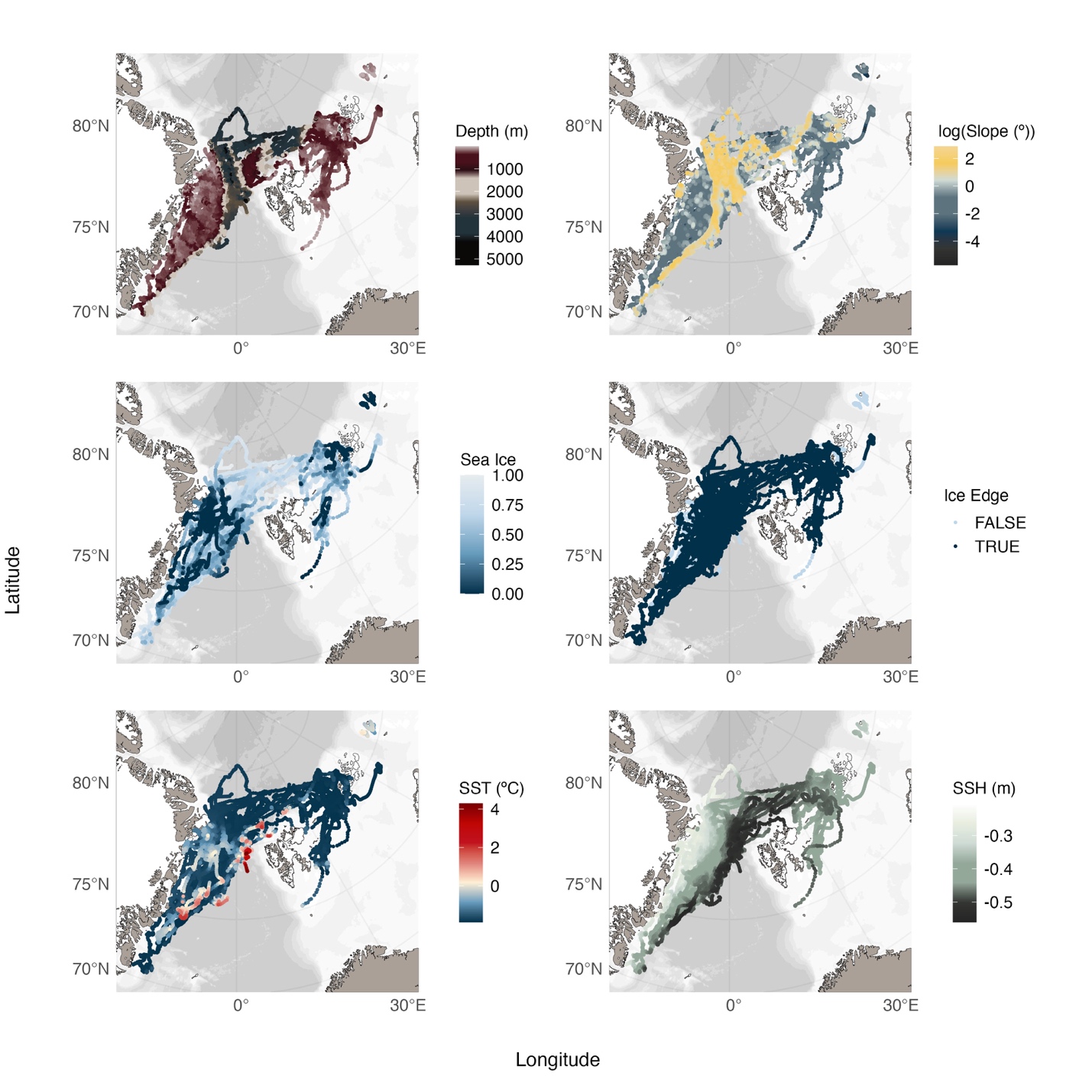


**Fig. S8** Maps of EGSB bowhead whale (n = 37) locations coloured by environmental conditions. Slope and sea surface temperature are plotted in increasing order to emphasize higher values, and sea ice concentration are plotted in decreasing order to emphasize lower values. Maps were produced using the R package *ggOceanMaps*^10^.


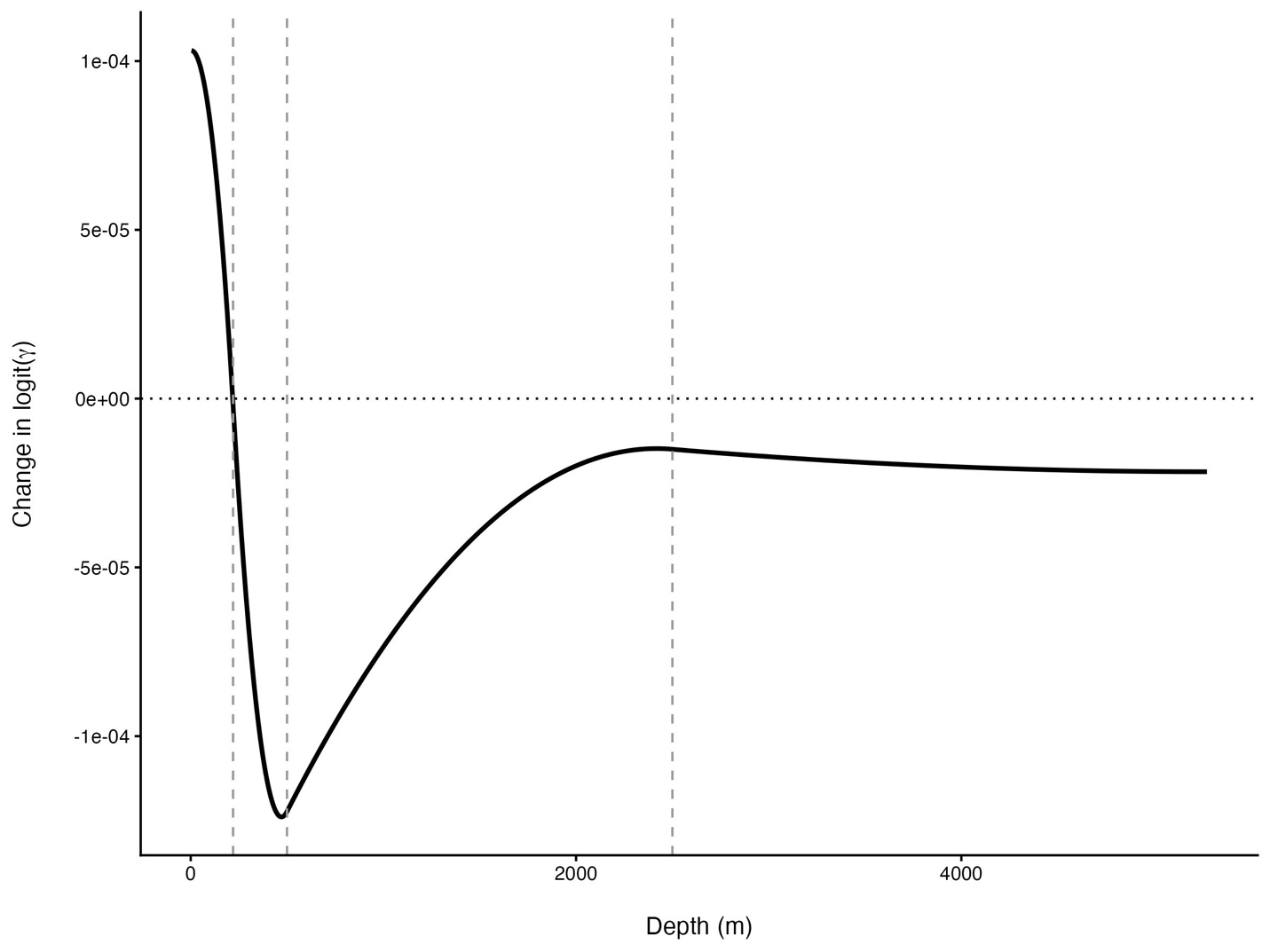


**Fig. S9** The first derivative (rate of change) of logit-transformed move persistence (*γ_t_*) in response to the natural cubic spline of depth (m) from the linear mixed-effects model. The horizontal grey dotted line indicates zero, where positive values represent an increase in move persistence and negative values represent a decrease in move persistence. Grey dashed lines indicate topographical knots used in the natural cubic spline (220 m, 500 m, 2500 m).


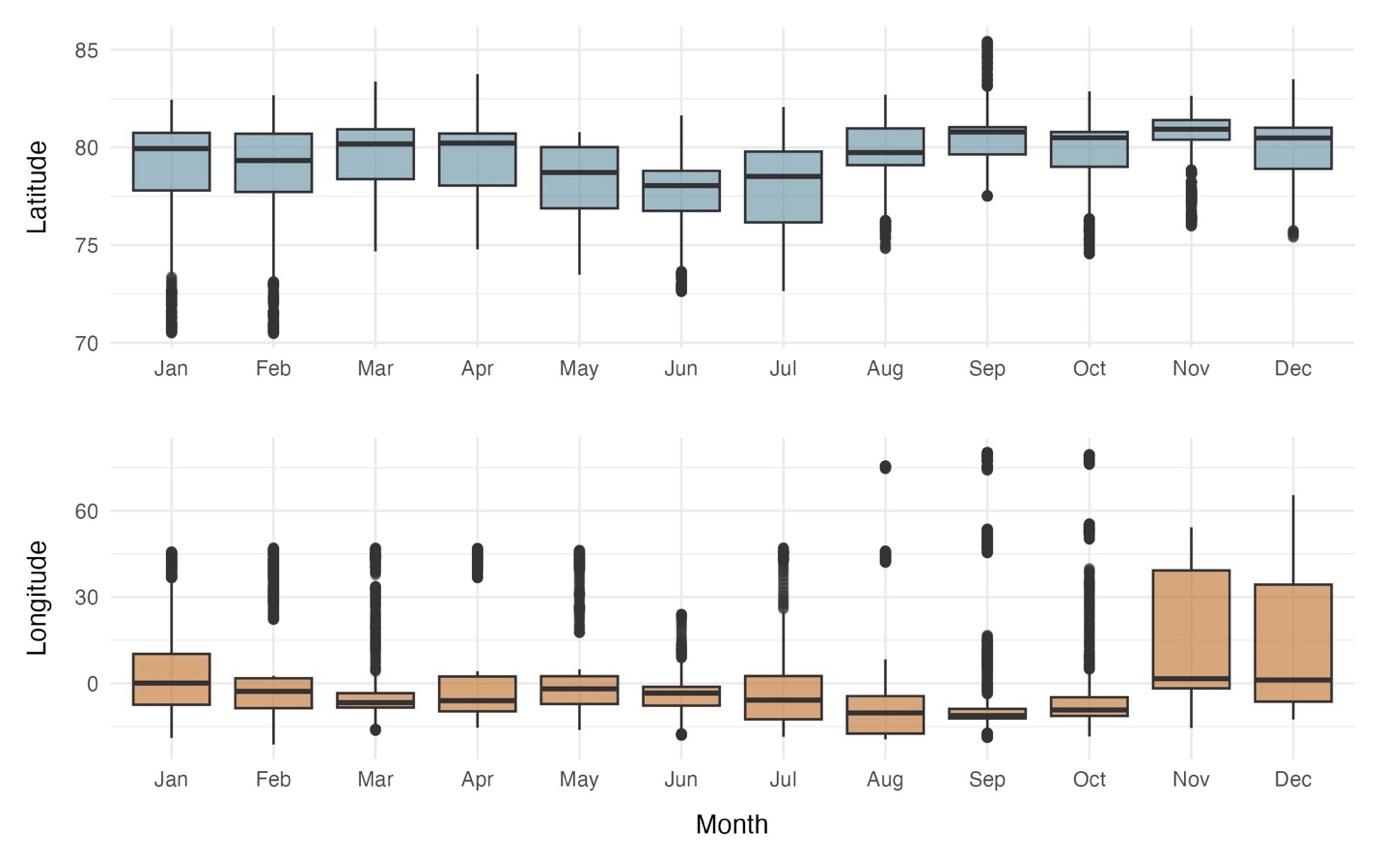


**Fig. S10** Boxplots for latitude (º) and longitude (º) of EGSB bowhead whale locations (n = 37) by month.

**REFERENCES**

1 Chambault, P. *et al.* Sea surface temperature predicts the movements of an Arctic cetacean: the bowhead whale. *Sci. Rep.* **8**, 9658 (2018).

2 Freitas, C., Lydersen, C., Fedak, M. A. & Kovacs, K. M. A simple new algorithm to filter marine mammal Argos locations. *Mar. Mammal Sci.* **24**, 315-325 (2008).

3 Pante, E., Simon-Bouhet, B., & Irisson, J. marmap: import, plot and analyze bathymetric and topographic data (2023).

4 Dorman, M. nngeo: k-Nearest Neighbor Join for Spatial Data (2024).

5 van Etten, J. gdistance: Distances and routes on geographical grids (2017).

6 Jonsen, I. D. *et al.* aniMotum, an R package for animal movement data: rapid quality control, behavioural estimation and simulation. *Methods Ecol. Evol.* **14**, 806-816 (2023).

7 Citta, J. J. *et al.* Influence of oceanography on bowhead whale (*Balaena mysticetus*) foraging in the Chukchi Sea as inferred from animal-borne instrumentation. *Cont. Shelf Res.* **224**, 104434 (2021).

8 Blackwell, S. B., Tervo, O. M., Lemming, N. E., Quakenbush, L. T. & Heide-Jørgensen, M. P. Drift dives in a bowhead whale (*Balaena mysticetus*). *Aquat. Mamm.* **48**, 656-660 (2022).

9 Fortune, S. M., Trites, A. W., LeMay, V., Baumgartner, M. F. & Ferguson, S. H. Year-round foraging across large spatial scales suggest that bowhead whales have the potential to adapt to climate change. *Front. Mar. Sci.* **9**, 853525 (2023).

10 Vihtakari, M. ggOceanMaps: Plot data on oceanographic maps using 'ggplot2' v. 2.2.0 (2024).
